# Supplementary material for: Probiotics, synbiotics and berberine in Type 2 diabetes mellitus: A systematic review, meta-analysis, and molecular dynamics simulation study
Source: PLoS One. 2026 May 29;21(5):e0348907. doi: 10.1371/journal.pone.0348907 (PMC13221027; doi:10.1371/journal.pone.0348907)
Supplement: S1 File — This file includes funnel plots, subgroup analyses, timeline and bubble plots, and molecular docking visualizations (Fig S1–S7). (DOCX) [file pone.0348907.s001.docx]

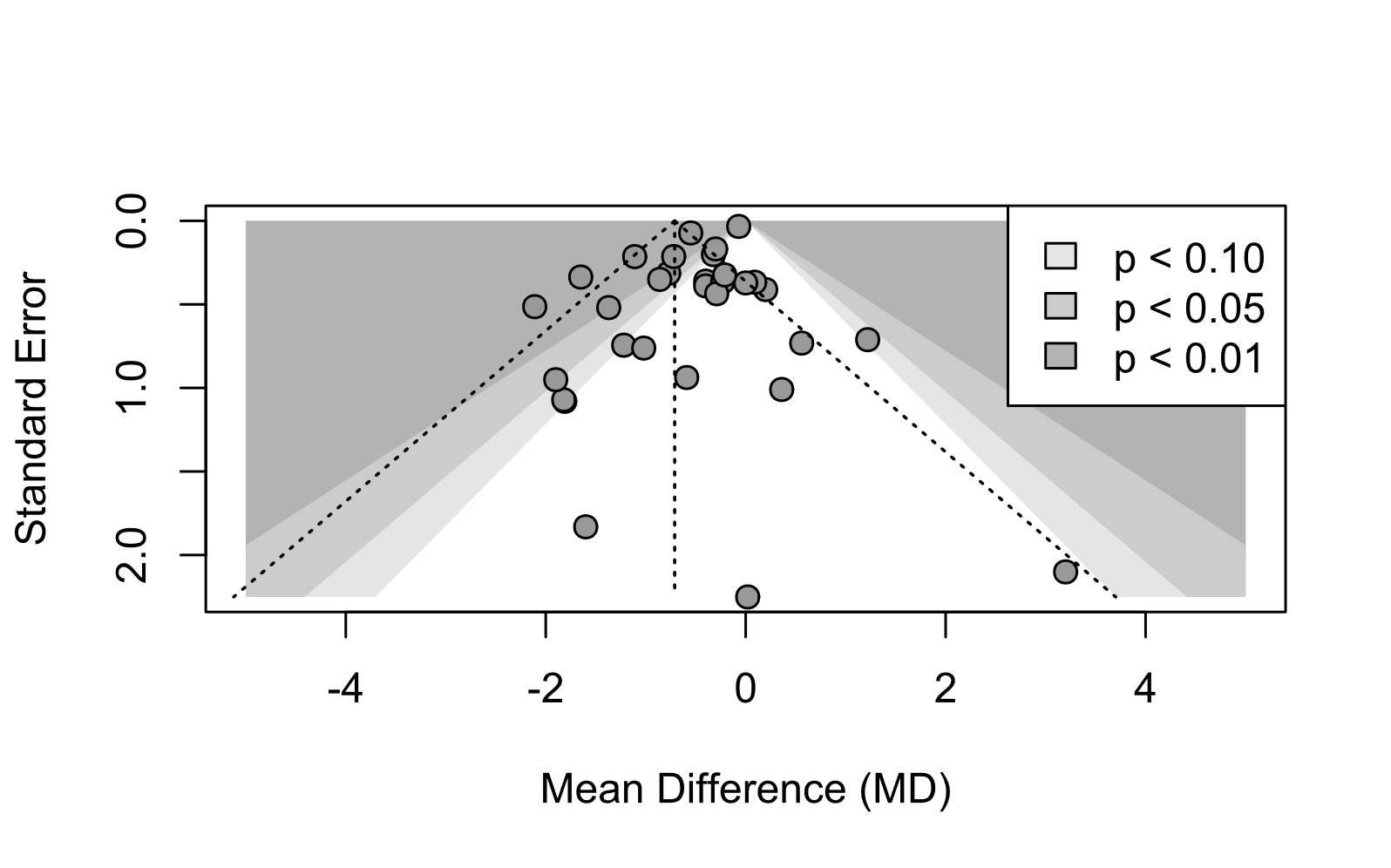


**Figure S1**: The funnel plot of FPG.


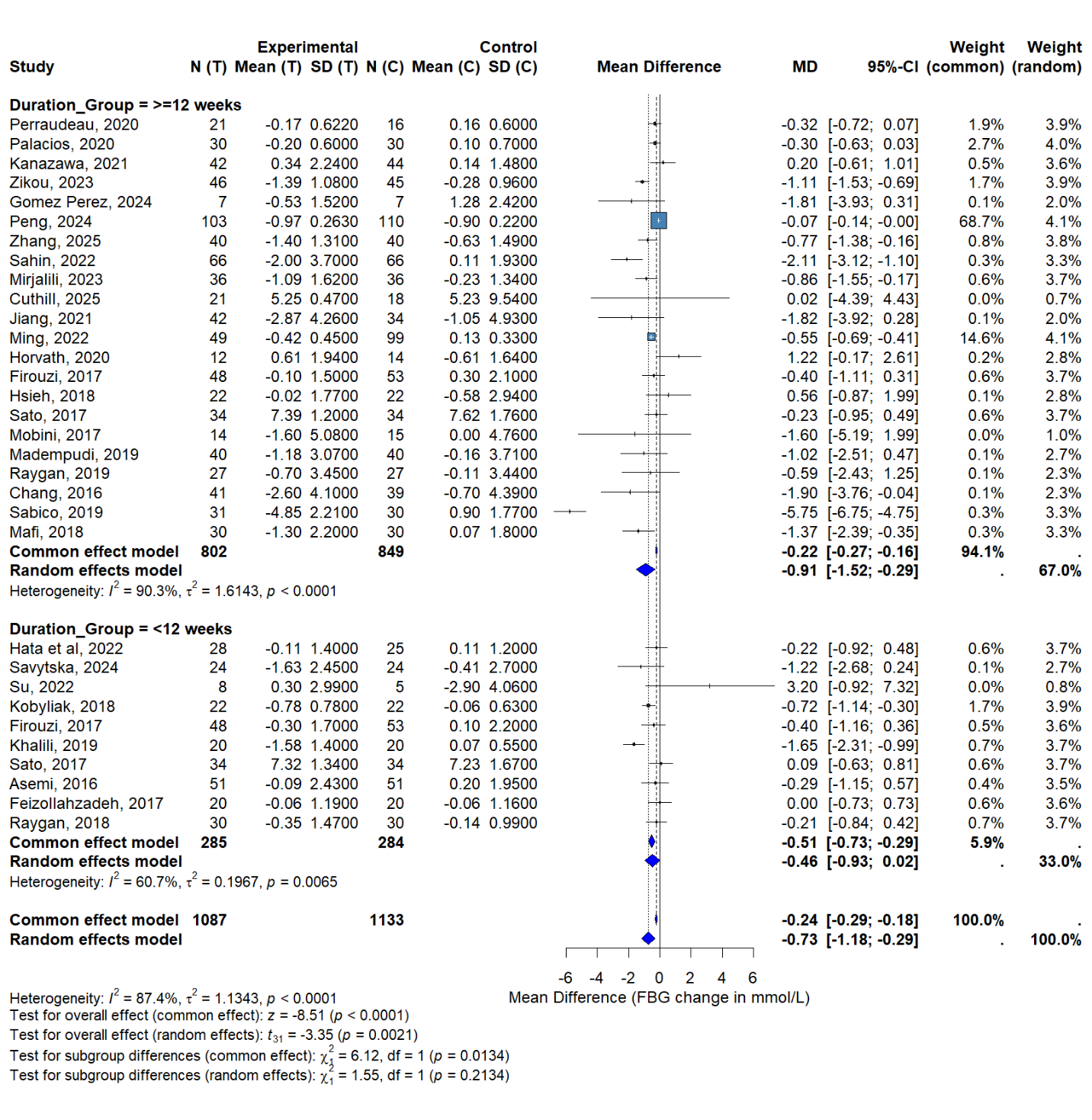


**Figure S2:** Meta-analysis of the subgroup of <12 and >= 12 weeks treatment of probiotics and BBR on FPG.

| **(a)** | **(b)** |
| --- | --- |
| **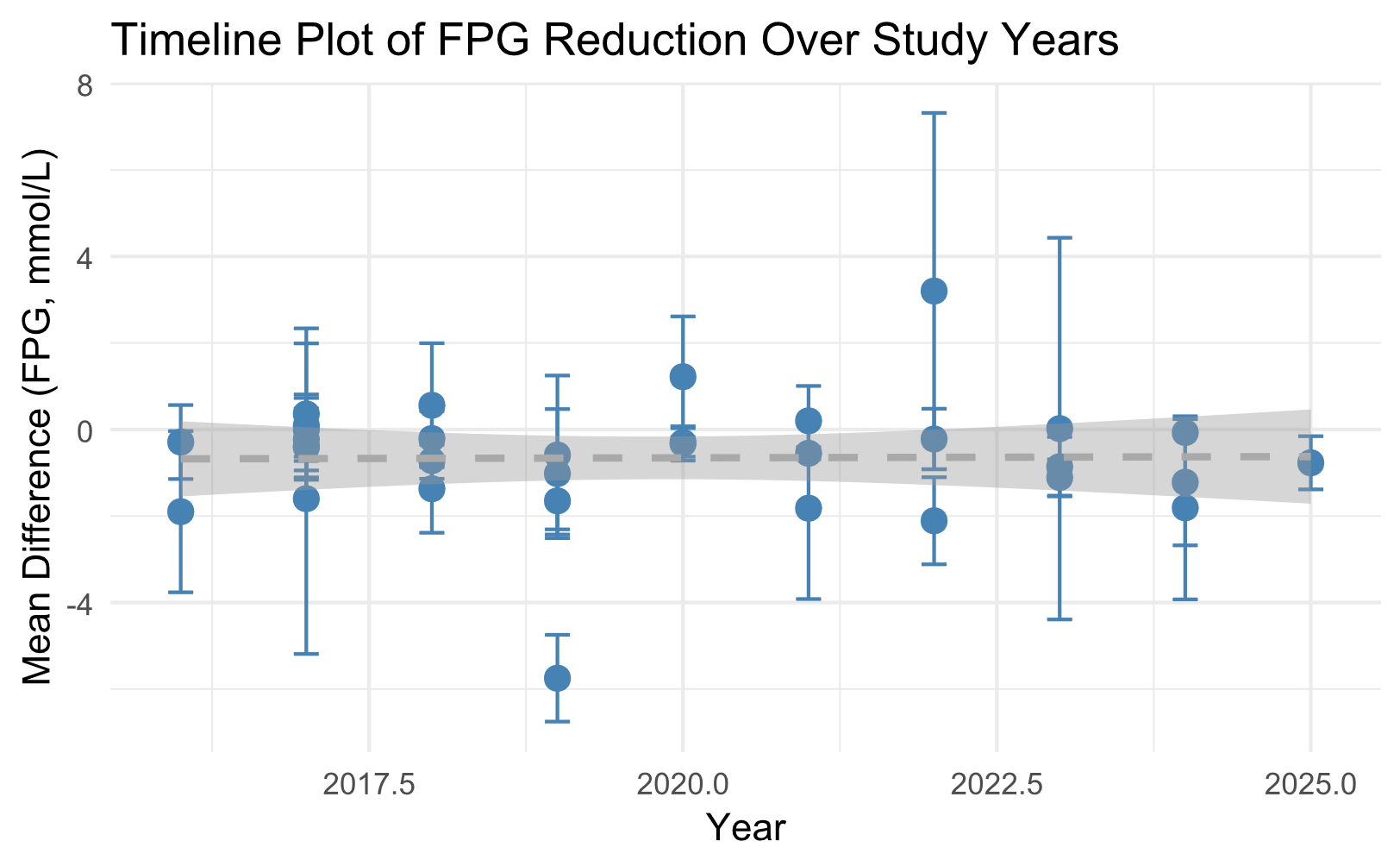** | **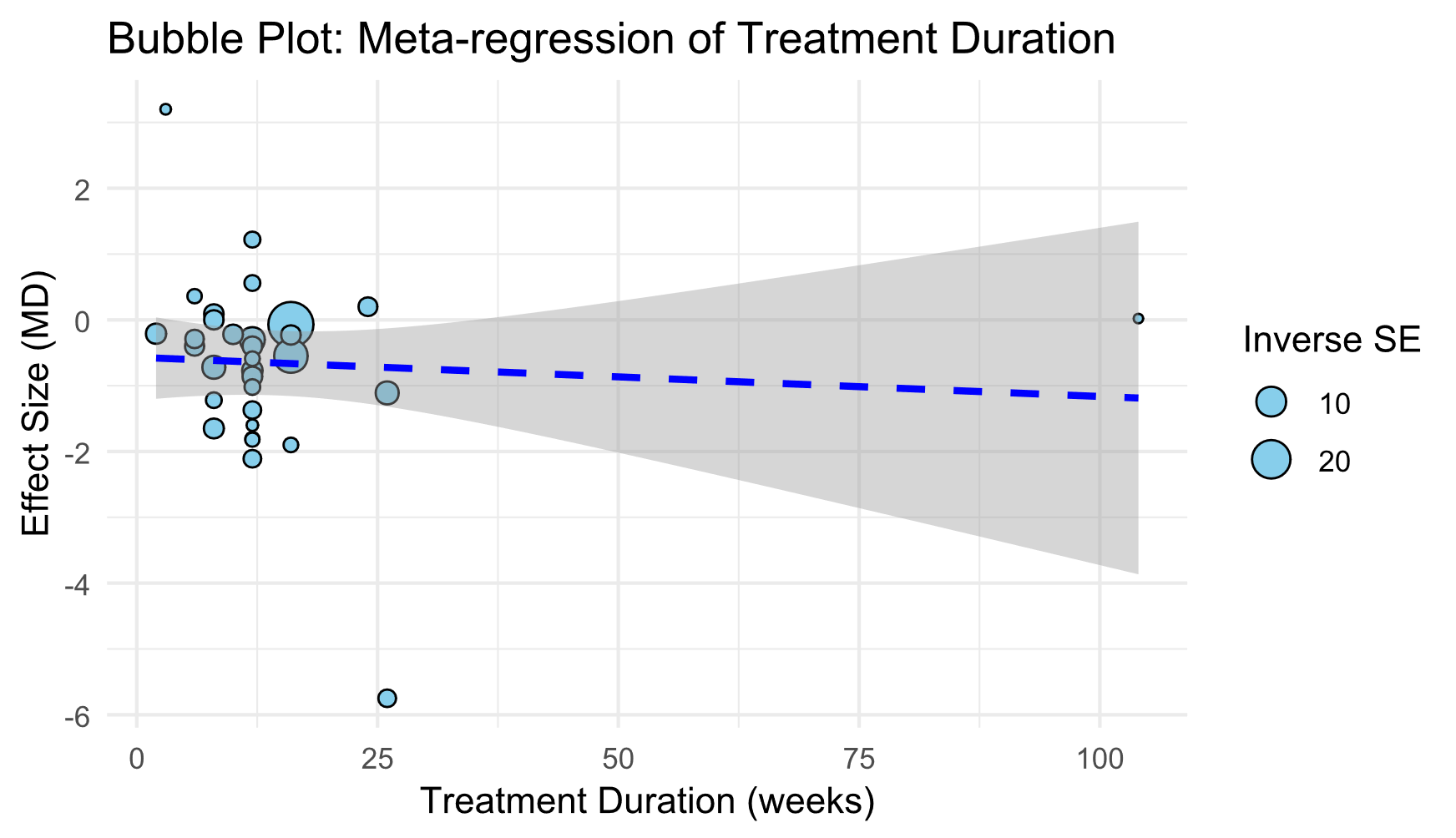** |

**Figure S3:** The timeline and bubble plot of FPG


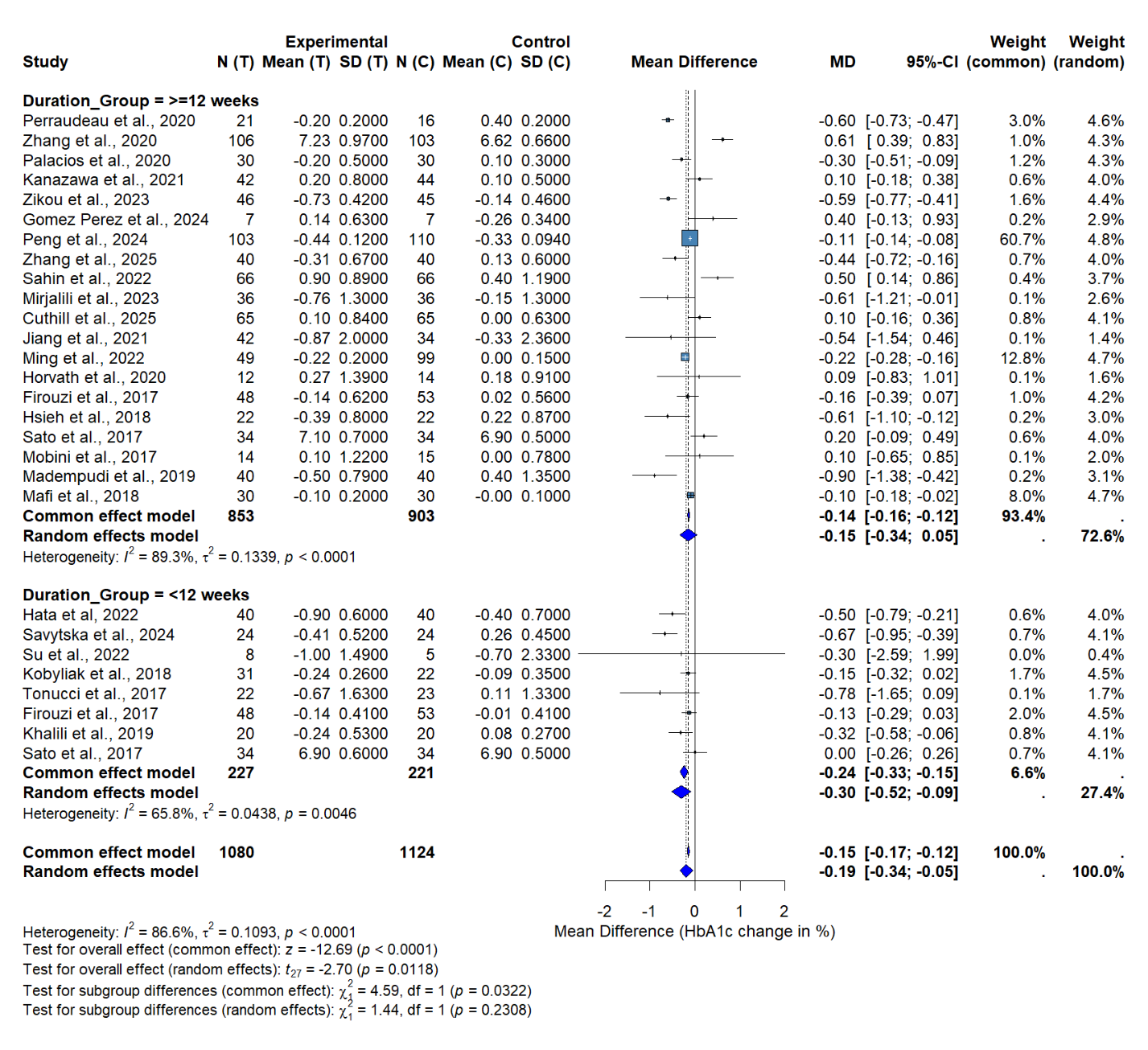


**Figure S4:** Meta-analysis of the subgroup of <12 and >= 12 weeks treatment of probiotics and BBR on HbA1c.

| **(a)** | **(b)** |
| --- | --- |
| 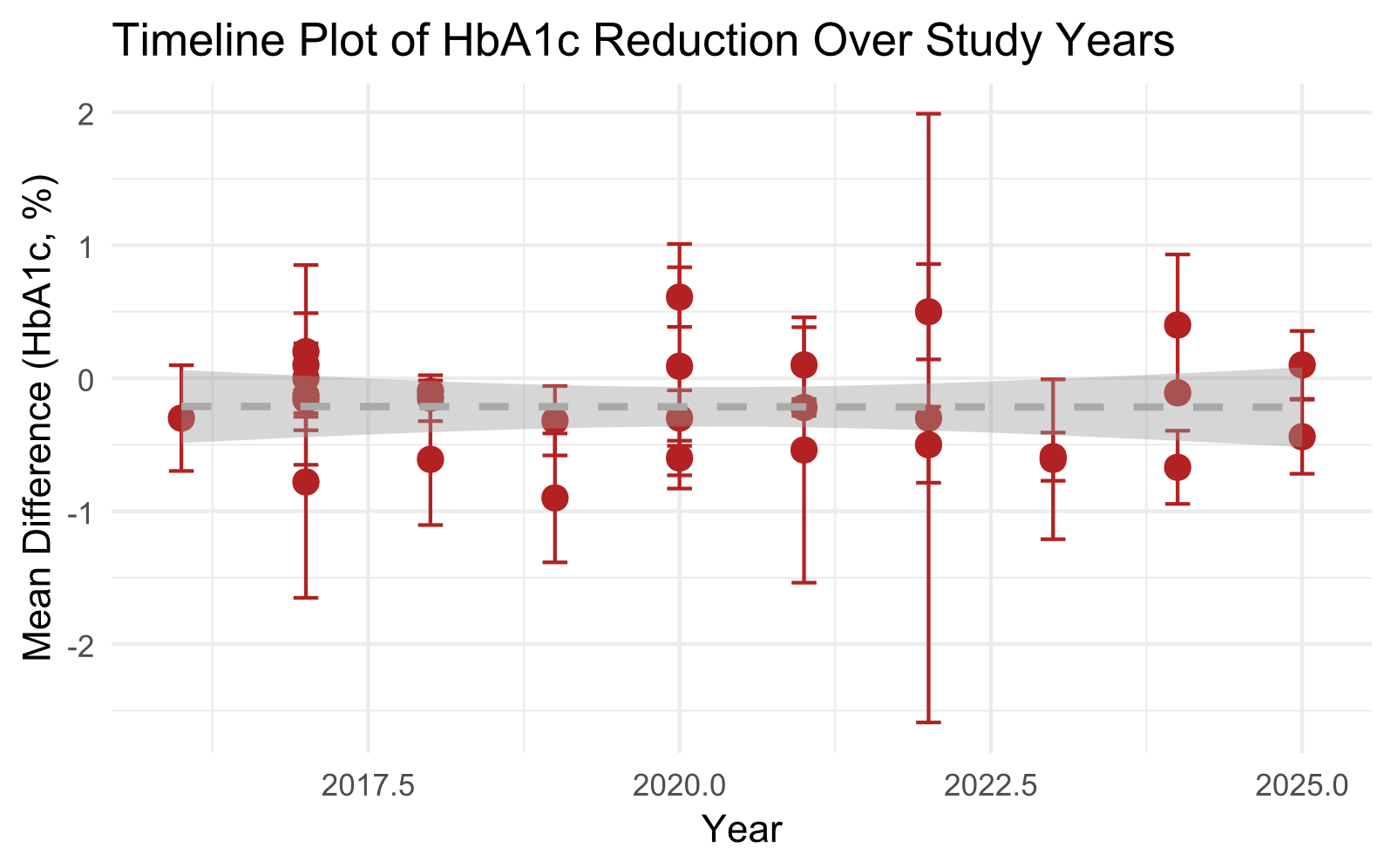 | 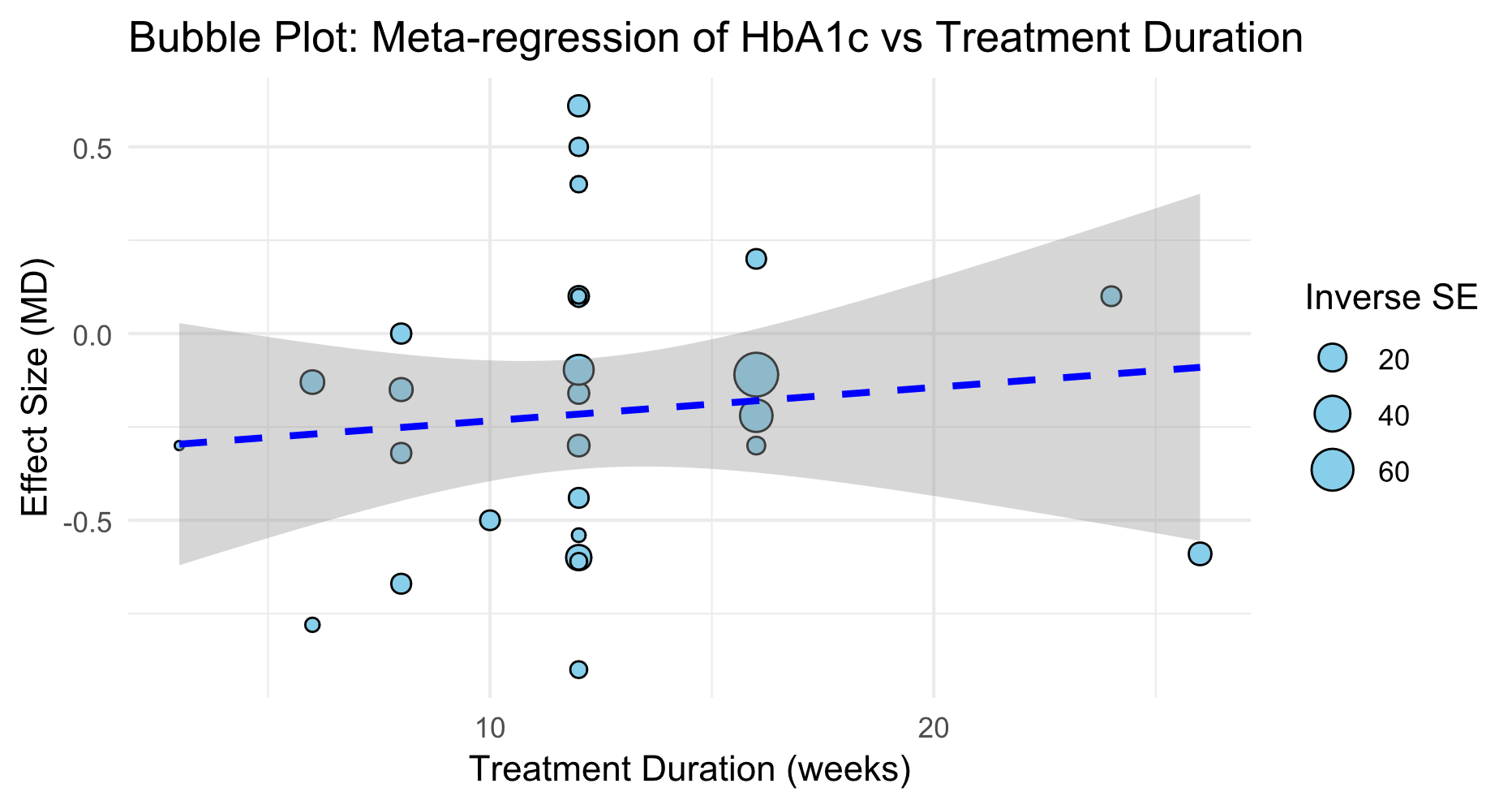 |

**Figure S5:** The timeline and bubble plot of HbA1c.


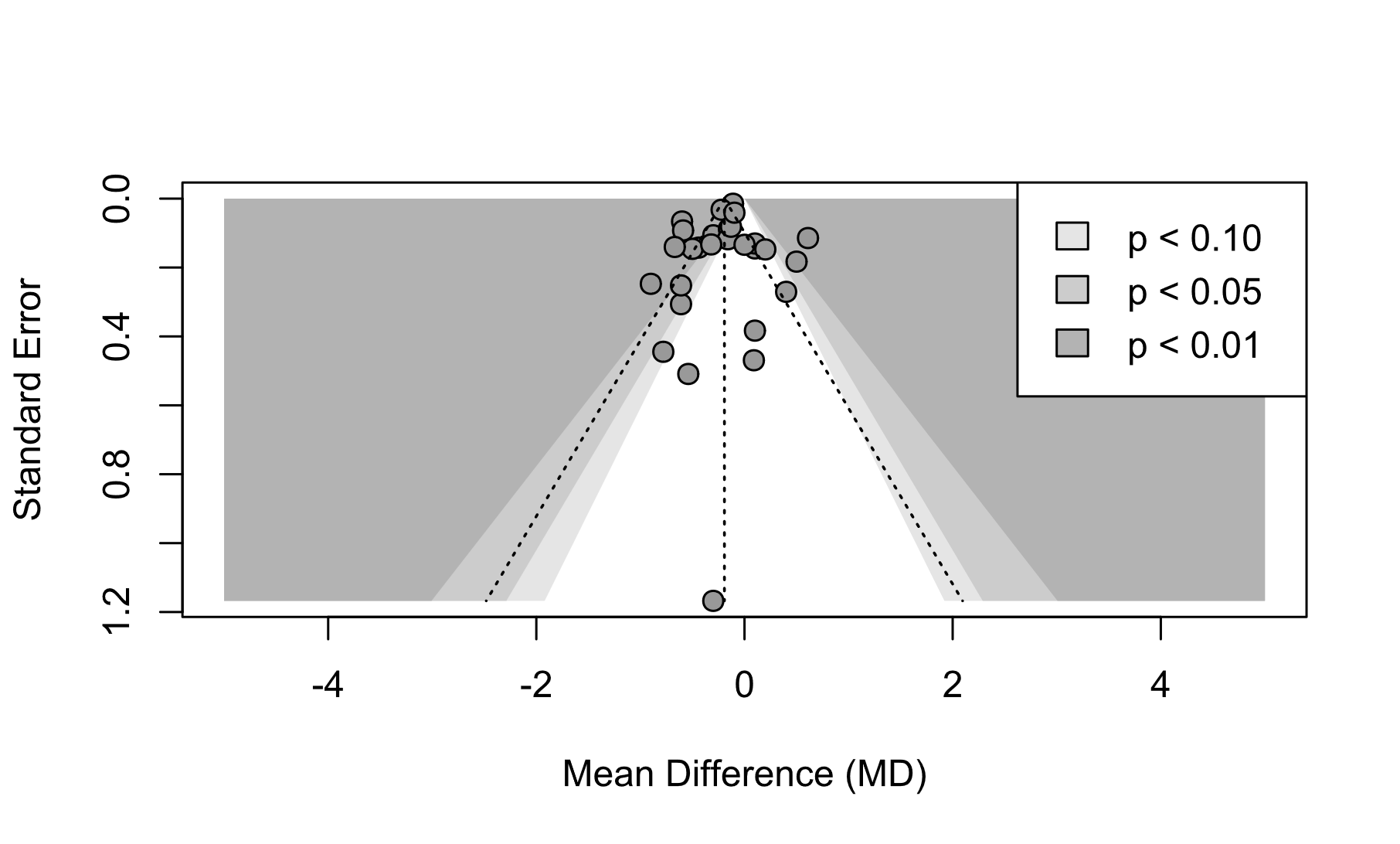


**Figure S6:** The funnel plot of HbA1c.


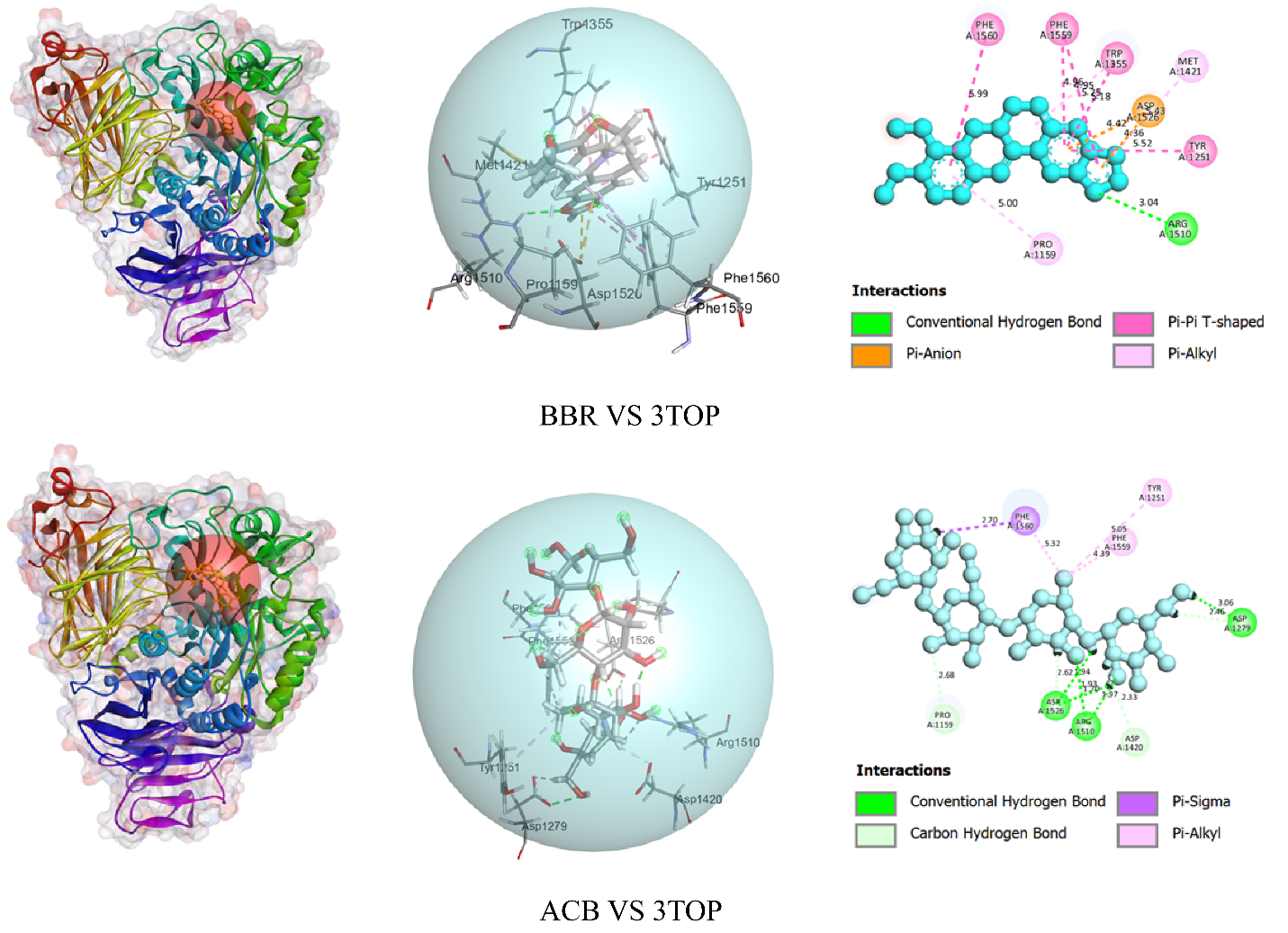


**Figure S7. Molecular docking analysis of berberine and acarbose with α-glucosidase, showing binding poses, interaction diagrams, and comparative overlay within the catalytic pocket.**
