# Supplementary material for: Probiotics, synbiotics and berberine in Type 2 diabetes mellitus: A systematic review, meta-analysis, and molecular dynamics simulation study
Source: PLoS One. 2026 May 29;21(5):e0348907. doi: 10.1371/journal.pone.0348907 (PMC13221027; doi:10.1371/journal.pone.0348907)
Supplement: S2 File — This file includes RMSD, RMSF, radius of gyration, SASA, hydrogen bond analysis, binding free energy calculations, and pharmacokinetic/toxicity predictions. (DOCX) [file pone.0348907.s002.docx]

**MD simulation Results**

The structural stability and dynamic behavior of the protein in its apo form and when bound to the standard and test compound were evaluated through molecular dynamics simulations. Four key structural parameters, root mean square deviation (RMSD), root mean square fluctuation (RMSF), radius of gyration (Rg), and solvent-accessible surface area (SASA), along with hydrogen bond analysis were investigated over a 100 ns simulation period to assess conformational stability and binding efficiency (**Figure S8**).

The RMSD profiles demonstrated that all systems reached equilibrium within approximately 10 ns, followed by stable trajectories for the remainder of the simulation. The apo protein exhibited RMSD values in the range of ~1.9–2.2 Å, while the berberine- and acarbose-bound complexes stabilized around ~2.5–3.0 Å and ~2.2–2.7 Å, respectively, indicating overall structural stability with minimal conformational deviations. The average RMSD values were 2.06 ± 0.11 Å for the apo form, 2.74 ± 0.13 Å for the berberine complex, and 2.48 ± 0.29 Å for the acarbose complex. The apo protein displayed slightly lower RMSD values compared to the ligand-bound systems, suggesting greater structural stability in the unbound state. Both ligand-bound systems exhibited slightly higher RMSD values, which may reflect conformational adjustments associated with ligand binding. The RMSD values of the berberine complex were comparable to those of the acarbose complex, indicating stable binding without major structural destabilization.

Residue-level flexibility was assessed through RMSF analysis, which demonstrated similar overall patterns across the three systems. Fluctuations were primarily confined to loop and terminal regions. The apo protein exhibited lower overall flexibility (0.85 ± 0.48 Å) compared with the ligand-bound systems, while the berberine (1.04 ± 1.03 Å) and acarbose (0.98 ± 0.80 Å) complexes showed slightly higher residue mobility. **The RMSF profiles of the berberine and acarbose complexes were largely overlapping, indicating comparable effects on residue-level dynamics.**

The compactness of the protein was evaluated using Rg, which remained relatively constant across all systems during the 100 ns simulation. The apo protein maintained a mean Rg of 29.00 ± 0.08 Å, while the berberine and acarbose complexes showed mean values of 29.03 ± 0.07 Å and 29.24 ± 0.12 Å, respectively. **The Rg trajectories of the two ligand-bound complexes largely overlapped, indicating similar effects on overall protein compactness.**

SASA analysis revealed differences in solvent exposure among the systems. The apo protein exhibited the lowest SASA value (340.28 ± 3.36 Å²), reflecting its compact structure. In contrast, the ligand-bound systems displayed higher SASA values, with means of 354.82 ± 4.68 Å² for berberine and 353.08 ± 4.62 Å² for acarbose. **The SASA profiles of the berberine and acarbose complexes were closely aligned, indicating comparable ligand-induced changes in solvent exposure.**

Principal component analysis (PCA) and free energy landscape (FEL) analyses showed that the apo protein sampled a relatively narrow conformational space, whereas ligand-bound systems exhibited broader distributions. The acarbose complex formed a compact and well-defined energy basin, whereas the berberine complex displayed multiple shallow basins. **The acarbose complex showed lower dispersion along the principal components than the berberine complex, reflecting reduced conformational variability.**

Hydrogen bond analysis showed that the standard compound (acarbose) formed an average of 6.37 ± 1.35 hydrogen bonds throughout the simulation, whereas the test compound (berberine) formed only 0.05 ± 0.22 transient hydrogen bonds. This indicates that acarbose relies predominantly on hydrogen bonding, while berberine likely interacts mainly through hydrophobic and van der Waals forces.


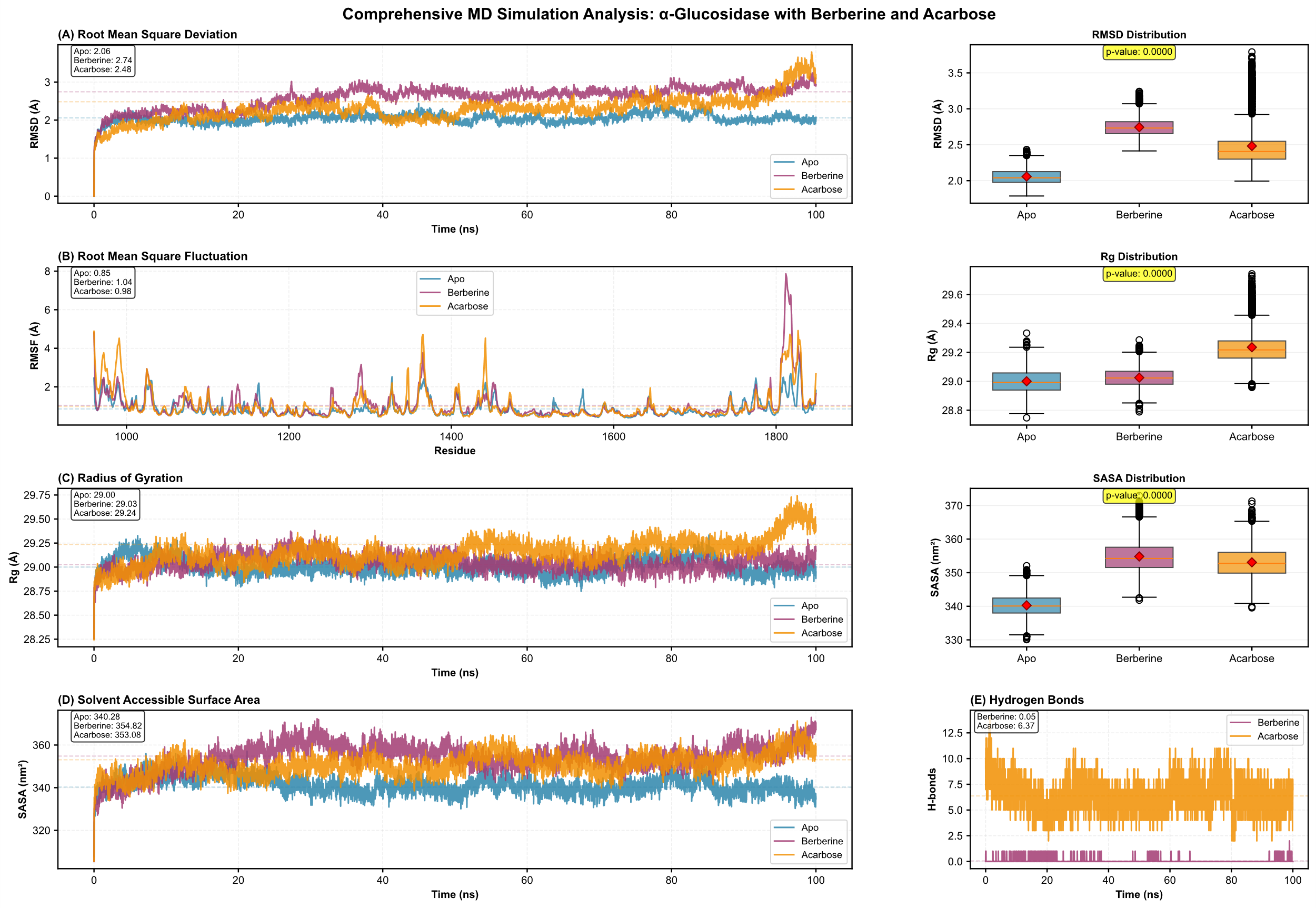
**Collectively, these findings suggest** that both ligands influence protein stability with comparable global effects on RMSD, Rg, SASA, and RMSF, while differing in their dominant interaction mechanisms, with acarbose exhibiting stronger hydrogen bonding and berberine relying primarily on non-polar interactions.

**Figure S8.** Structural stability and hydrogen bond analysis of α-glucosidase in apo and ligand-bound states during 100-ns MD simulations. (a), RMSD plots showing overall stability of apo, berberine-, and acarbose-bound complexes. (b), RMSF profiles indicating residue-level flexibility across systems. (c), Radius of gyration (Rg) plots demonstrating protein compactness. (d), Solvent accessible surface area (SASA) trajectories showing solvent exposure differences. (e), Number of protein–ligand hydrogen bonds as a function of simulation time.

| (a) |
| --- |
| 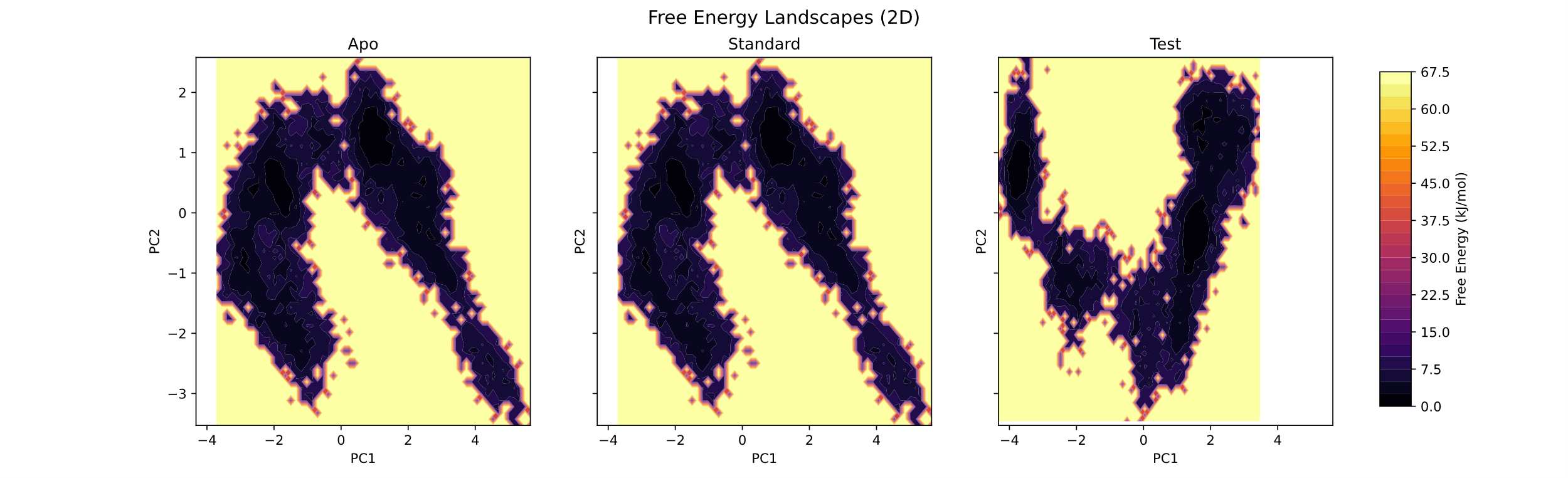 |
| (b) |
| 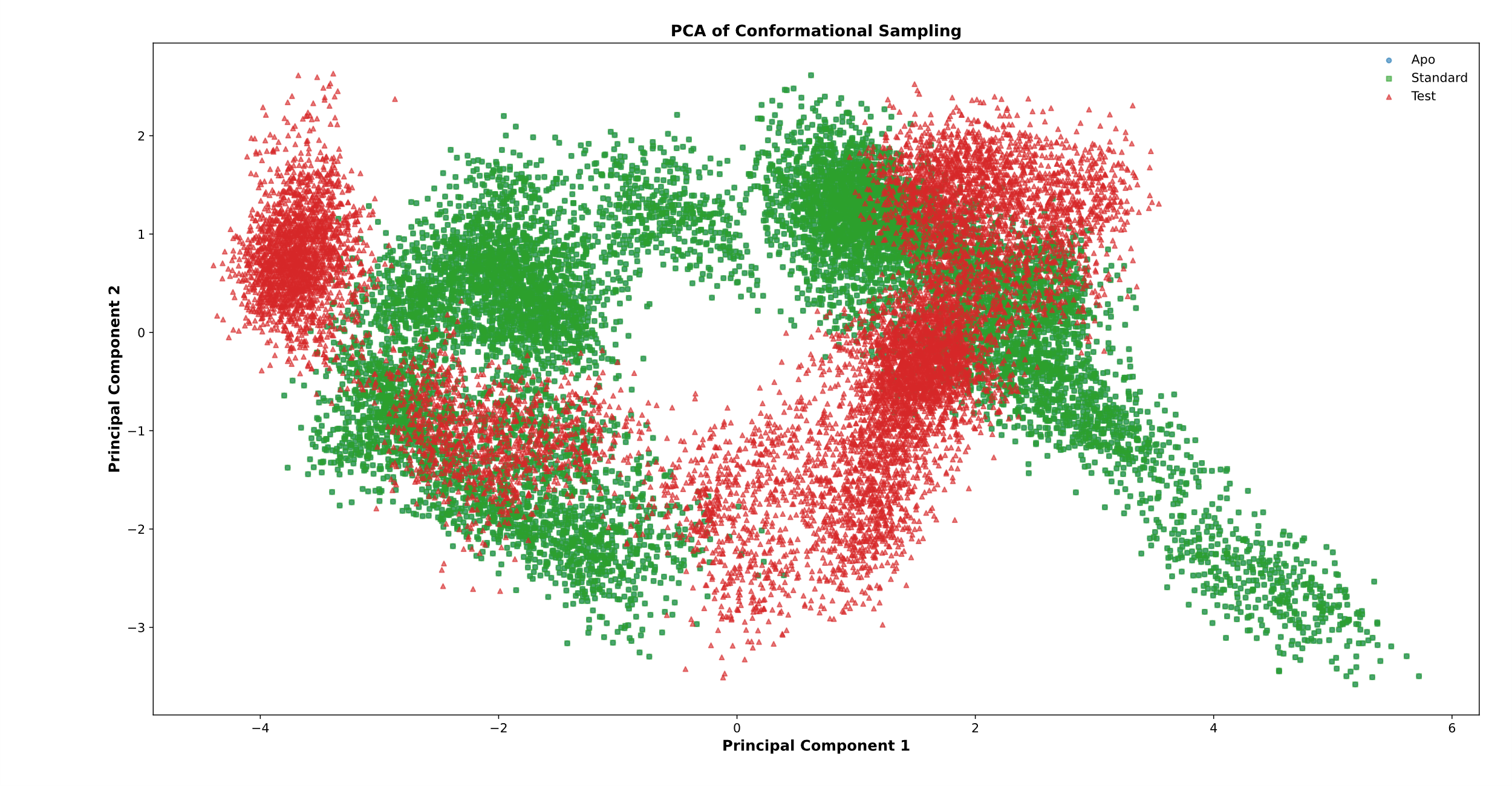 |

**Figure S9. a,** Principal component analysis (PCA) and **b,** free energy landscape (FEL) of α-glucosidase in apo, acarbose-bound, and berberine-bound states over 100 ns MD simulations. The FELs were constructed using the first two principal components (PC1 and PC2), showing conformational sampling and stability differences among the systems.

**MM-PBSA Binding Free Energy Analysis**

The binding affinities of the protein–ligand complexes were further evaluated using MM-PBSA calculations over 100 ns of simulation. The results indicated that the standard ligand (acarbose) exhibited a substantially more favorable binding free energy compared to the test compound (berberine). As shown in **Table S1**, the total binding free energy (ΔG_bind) of the acarbose complex was −25.66 ± 6.15 kcal·mol⁻¹, whereas berberine displayed (**Table S3**) a markedly less favorable value of −6.35 ± 7.01 kcal·mol⁻¹.

Component energy decomposition revealed that, for both ligands, strong electrostatic (EEL) and van der Waals (VDWAALS) interactions in the gas phase were largely offset by unfavorable polar solvation energies (EPB). For the acarbose complex, van der Waals (−26.15 ± 5.68 kcal·mol⁻¹) and electrostatics (−501.84 ± 39.42 kcal·mol⁻¹) contributed significantly, with partial compensation from polar solvation (+506.68 ± 42.47 kcal·mol⁻¹). In contrast, the berberine complex showed weaker electrostatic stabilization (−376.98 ± 12.10 kcal·mol⁻¹) and a smaller non-polar solvation contribution (−3.11 ± 0.26 kcal·mol⁻¹), leading to its overall less favorable ΔG_bind. These values suggest that the balance of van der Waals and non-polar interactions contributes to the stronger binding affinity of acarbose.

Per-residue decomposition (**Figure S10)** identified several residues with notable contributions to ligand binding. Residues such as ARG1510, TRP1523, PRO1159, and PHE1560 stabilized the acarbose complex, while certain acidic residues (e.g., ASP1420, ASP1555) contributed unfavorably due to desolvation penalties. Differential analysis between the two ligands highlighted that acarbose established stronger interactions with catalytic and binding-site residues compared to berberine, which relied primarily on transient hydrophobic contacts (**Table S2 & S4**).

Overall, the MM-PBSA results are consistent with the dynamic analyses and hydrogen bond profiles, indicating stronger and more stable interactions for acarbose compared with berberine.

**Table S1.** MM-PBSA binding free energy decomposition for acarbose complexes with α-glucosidase.

| Frames | VDWAALS | EEL | EPB | ENPOLAR | GGAS | GSOLV | TOTAL |
| --- | --- | --- | --- | --- | --- | --- | --- |
| Average | -26.15 | -501.84 | 506.68 | -4.35 | -527.99 | 502.33 | -25.66 |
| SD | 5.68 | 39.42 | 42.47 | 0.47 | 41.48 | 42.08 | 6.15 |
| SEM | 0.57 | 3.94 | 4.25 | 0.05 | 4.15 | 4.21 | 0.61 |

**Table S2.** Top per-residue energy contributions from MM-PBSA for acarbose complexes.

| Frames | A: ASP: 1157 | A: PRO: 1159 | A: ASP: 1279 | A: ASP: 1420 | A: LYS: 1460 | A: ARG:1510 | A: TRP: 1523 | A: ASP: 1526 | A: ASP: 1555 | A: PHE: 1559 | A: PHE :1560 | A: ARG: 1582 | B: LIG: 1850 |
| --- | --- | --- | --- | --- | --- | --- | --- | --- | --- | --- | --- | --- | --- |
| Average | 1.73 | -1.67 | 0.92 | 4.66 | 2.15 | -2.25 | -1.43 | -0.93 | 4.7 | -0.79 | -1.03 | 1.24 | -23.51 |
| SD | 2.66 | 0.58 | 2.14 | 4.46 | 1.01 | 2.04 | 0.62 | 4.66 | 2.73 | 0.44 | 0.54 | 2.82 | 6.02 |
| SEM | 0.27 | 0.06 | 0.21 | 0.45 | 0.1 | 0.2 | 0.06 | 0.47 | 0.27 | 0.04 | 0.05 | 0.28 | 0.6 |

**Table S3.** MM-PBSA binding free energy decomposition for berberine complexes with α-glucosidase.

| Frames | VDWAALS | EEL | EPB | ENPOLAR | GGAS | GSOLV | TOTAL |
| --- | --- | --- | --- | --- | --- | --- | --- |
| Average | -26.63 | -376.98 | 400.36 | -3.11 | -403.6 | 397.25 | -6.35 |
| SD | 2.64 | 12.1 | 16.65 | 0.26 | 13.4 | 16.53 | 7.01 |
| SEM | 0.26 | 1.21 | 1.67 | 0.03 | 1.34 | 1.65 | 0.7 |

**Table S4.** Top per-residue energy contributions from MM-PBSA for berberine complexes.

| Frames | A:TYR  1251 | A:ASP:  1279 | A:ASP:  1420 | A:TRP:  1523 | A:ASP:  1526 | A:ASP:  1555 | A:PHE:  1559 | A:ARG:  1582 | B:LIG:  1850 |
| --- | --- | --- | --- | --- | --- | --- | --- | --- | --- |
| Average | -1.33 | 1.13 | 3.12 | -0.63 | 3.63 | 4.89 | -1.56 | 2.34 | -10.9 |
| SD | 0.76 | 2.65 | 3.61 | 0.39 | 2.37 | 1.73 | 0.43 | 2.44 | 2.17 |
| SEM | 0.08 | 0.26 | 0.36 | 0.04 | 0.24 | 0.17 | 0.04 | 0.24 | 0.22 |

| **Test** | **Standard** |
| --- | --- |
| **a** | **b** |
| 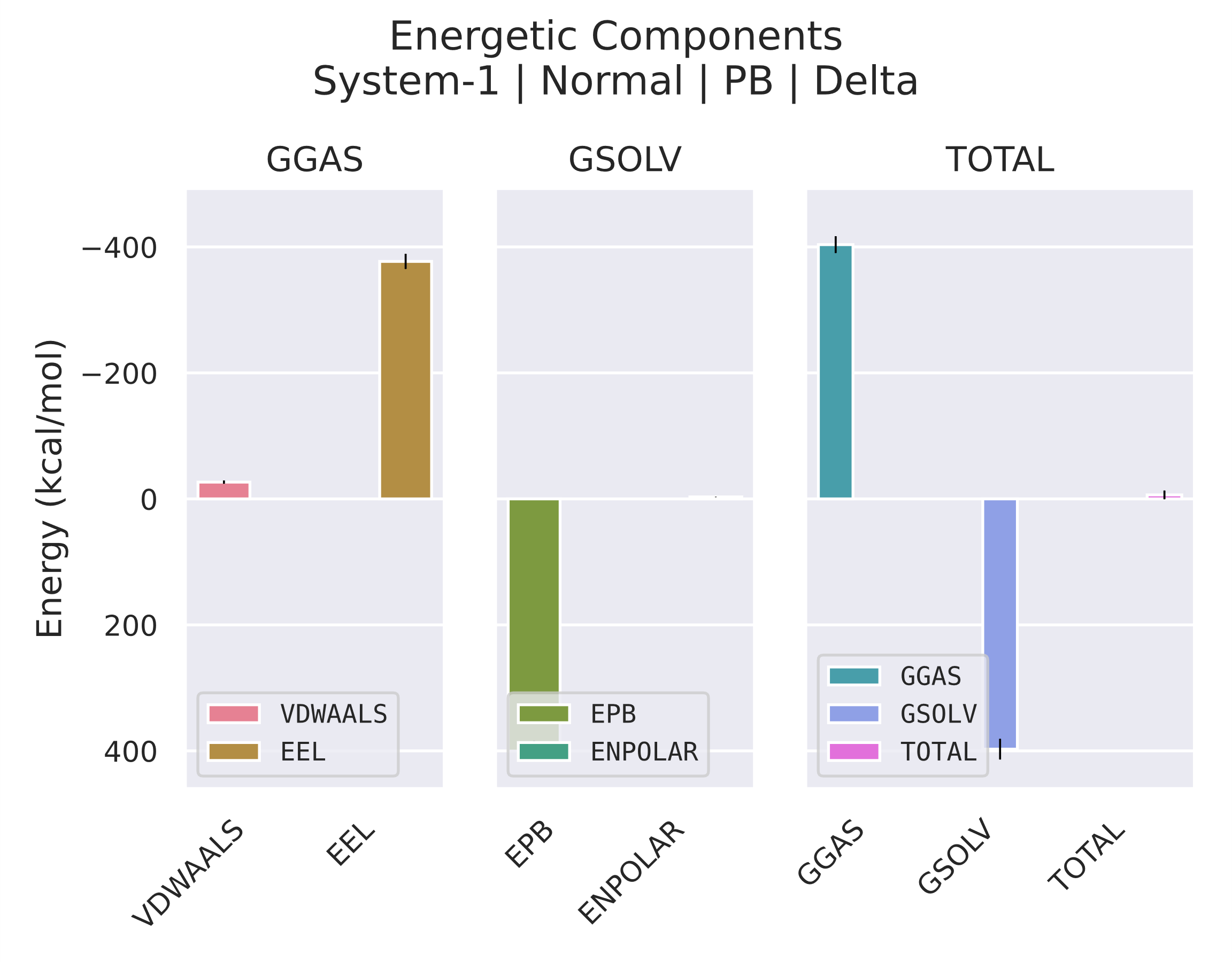 | 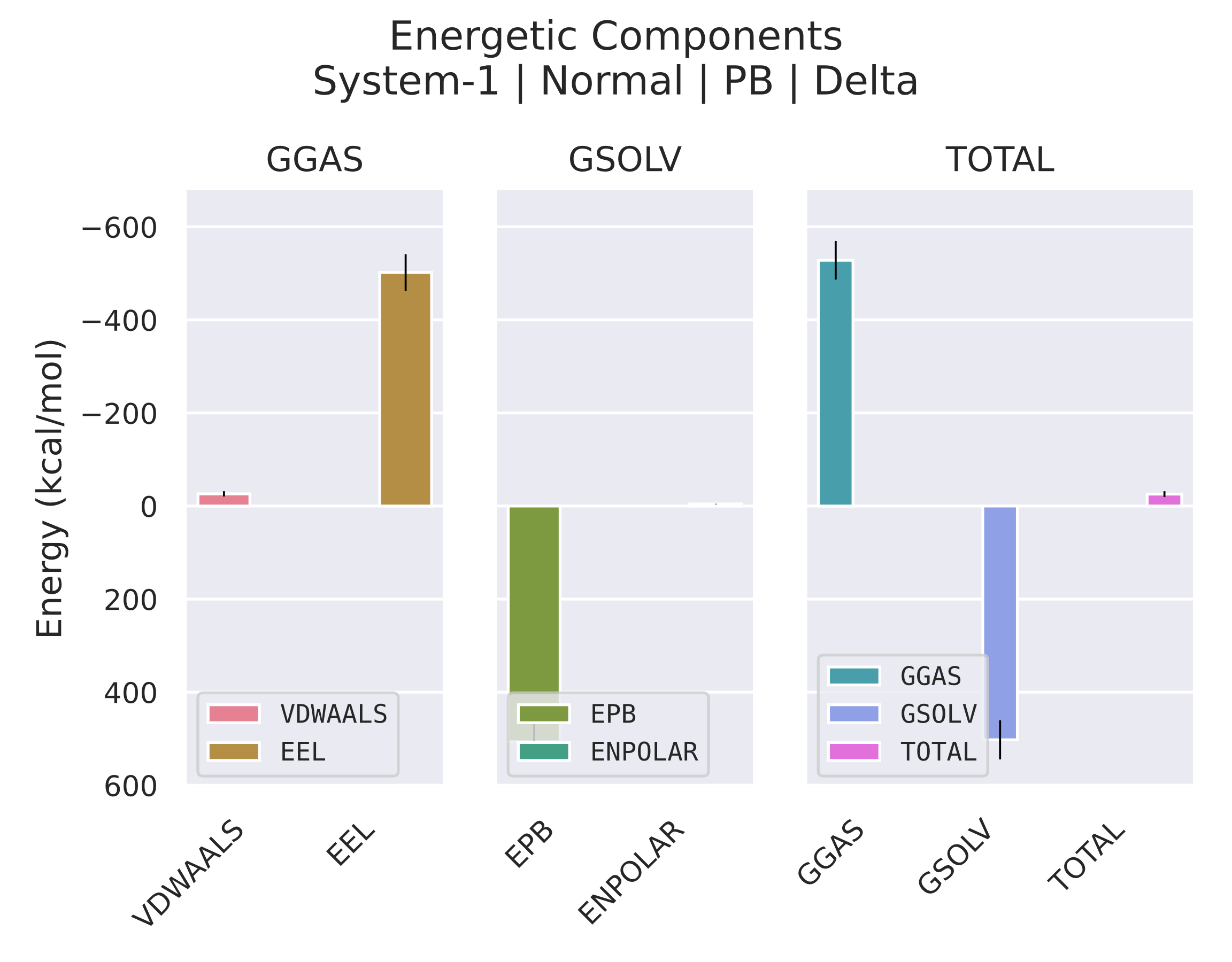 |
| **c** | **d** |
| 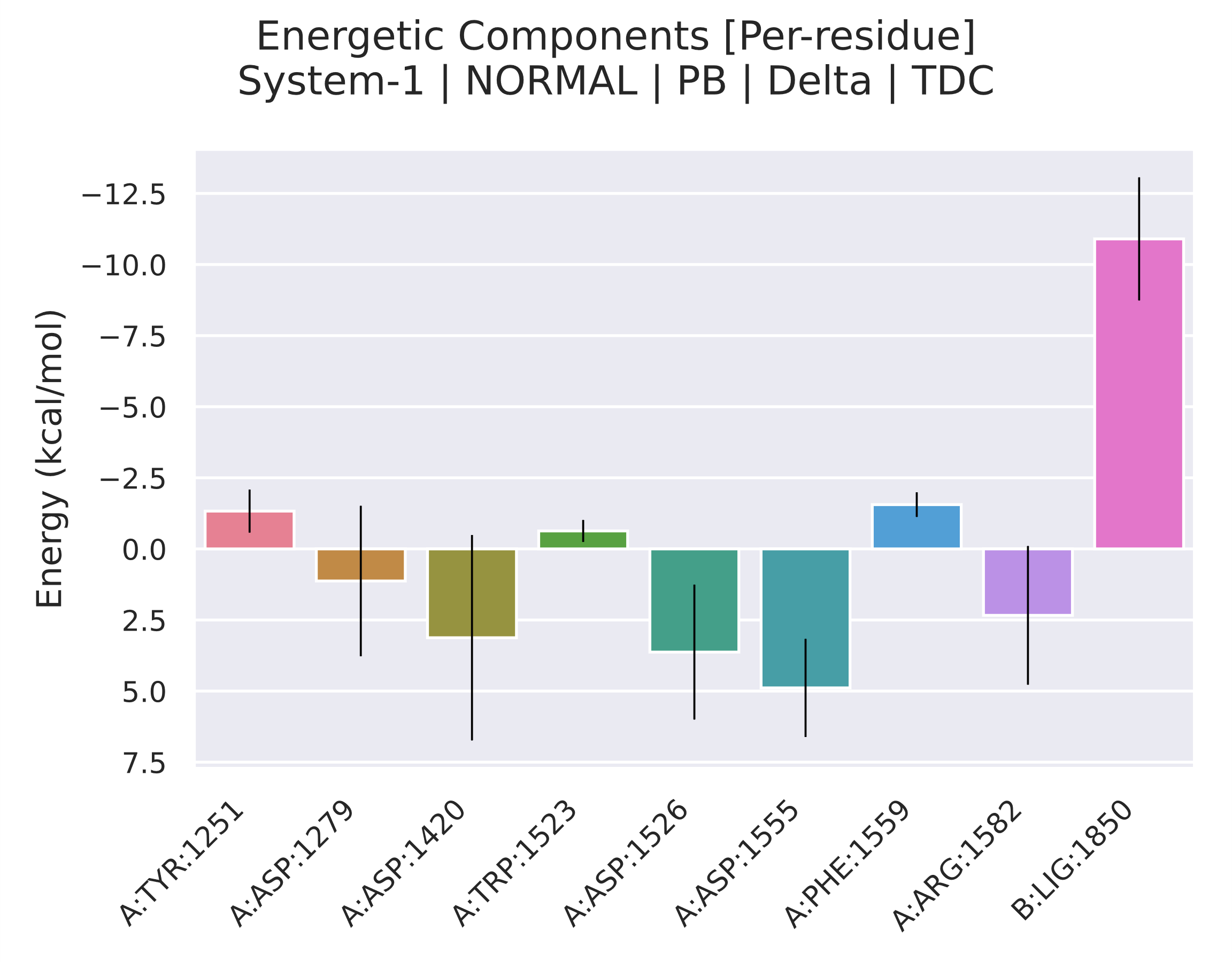 | 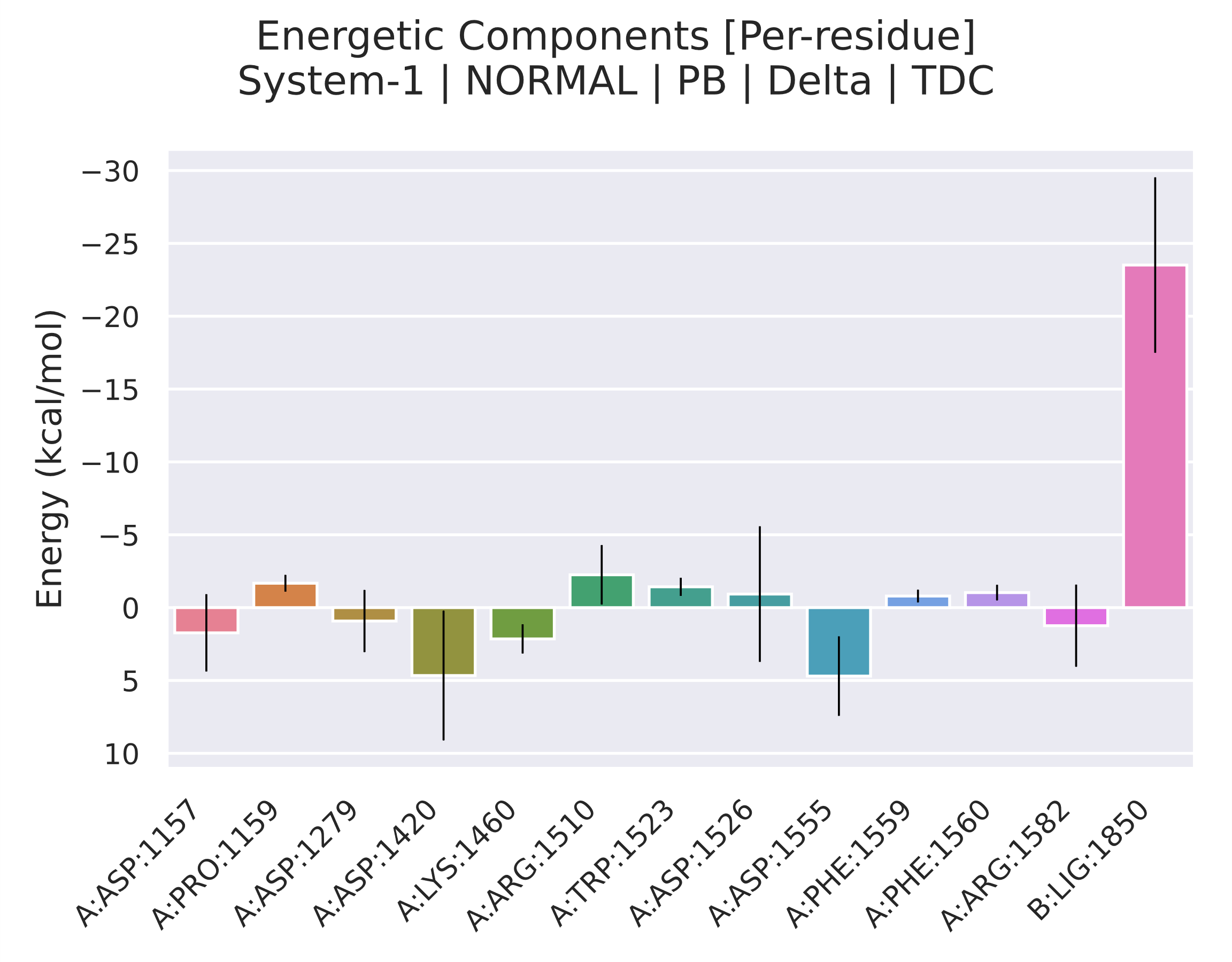 |
| **e** | **f** |
| 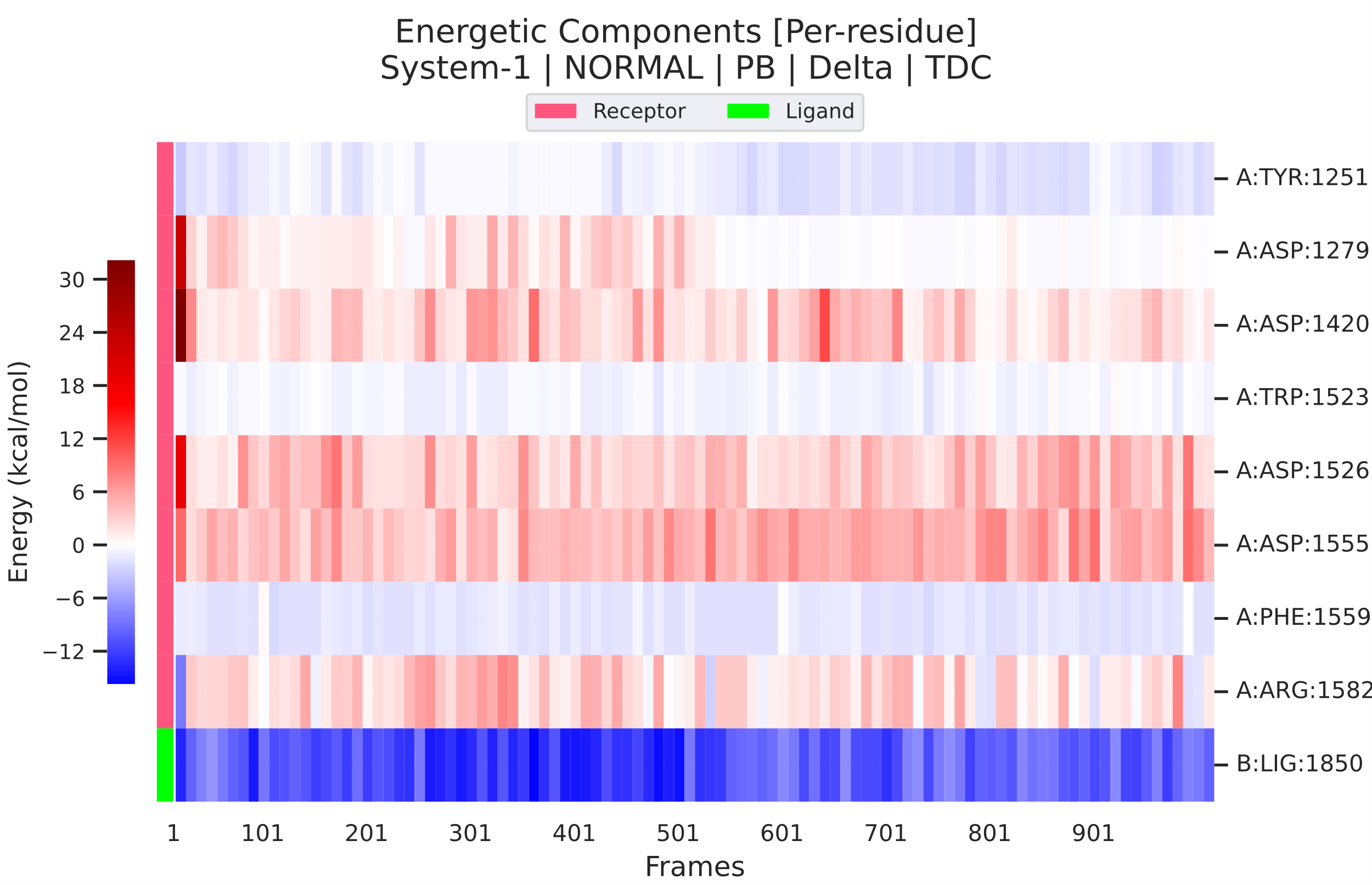 | 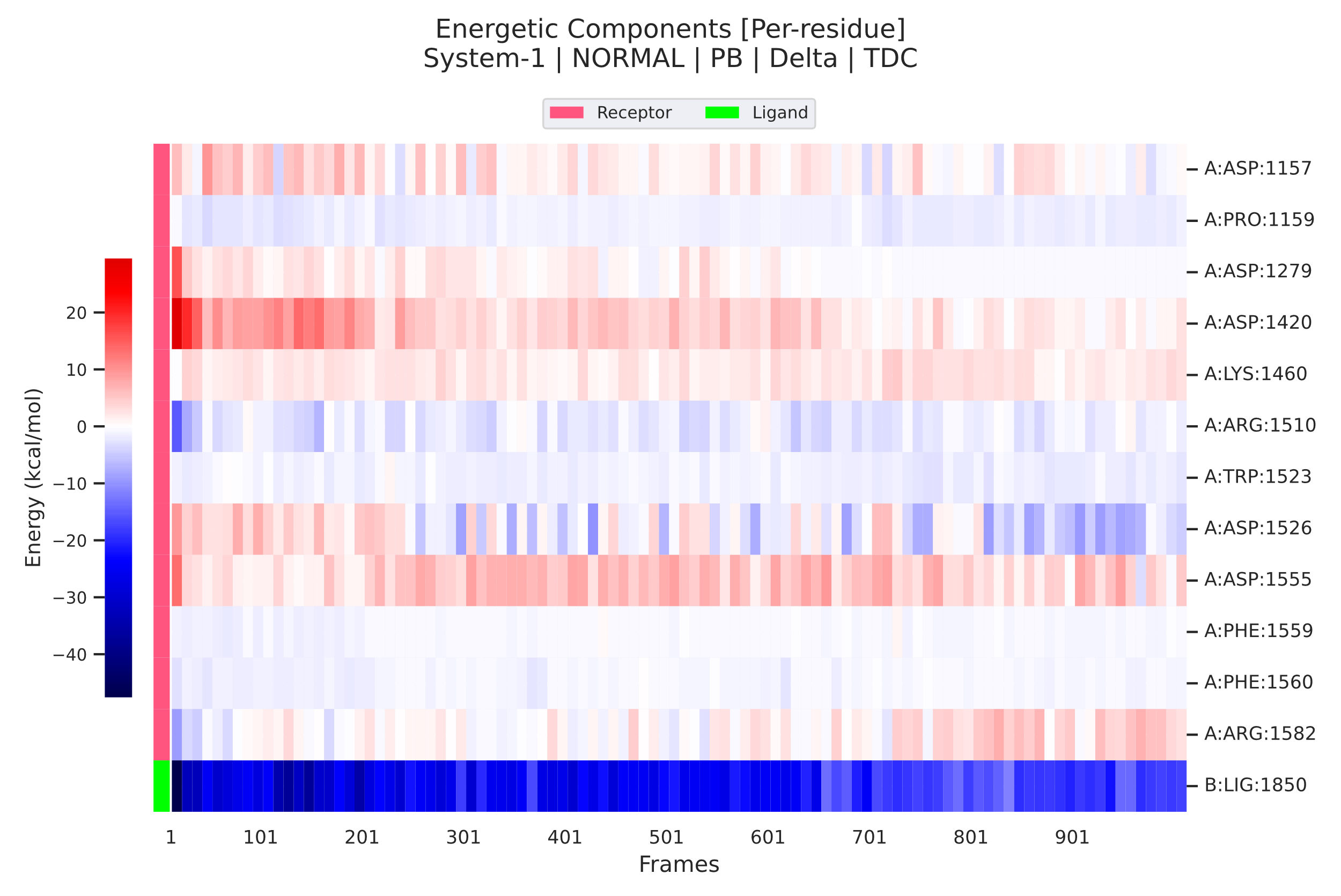 |
| **g** | **h** |
| 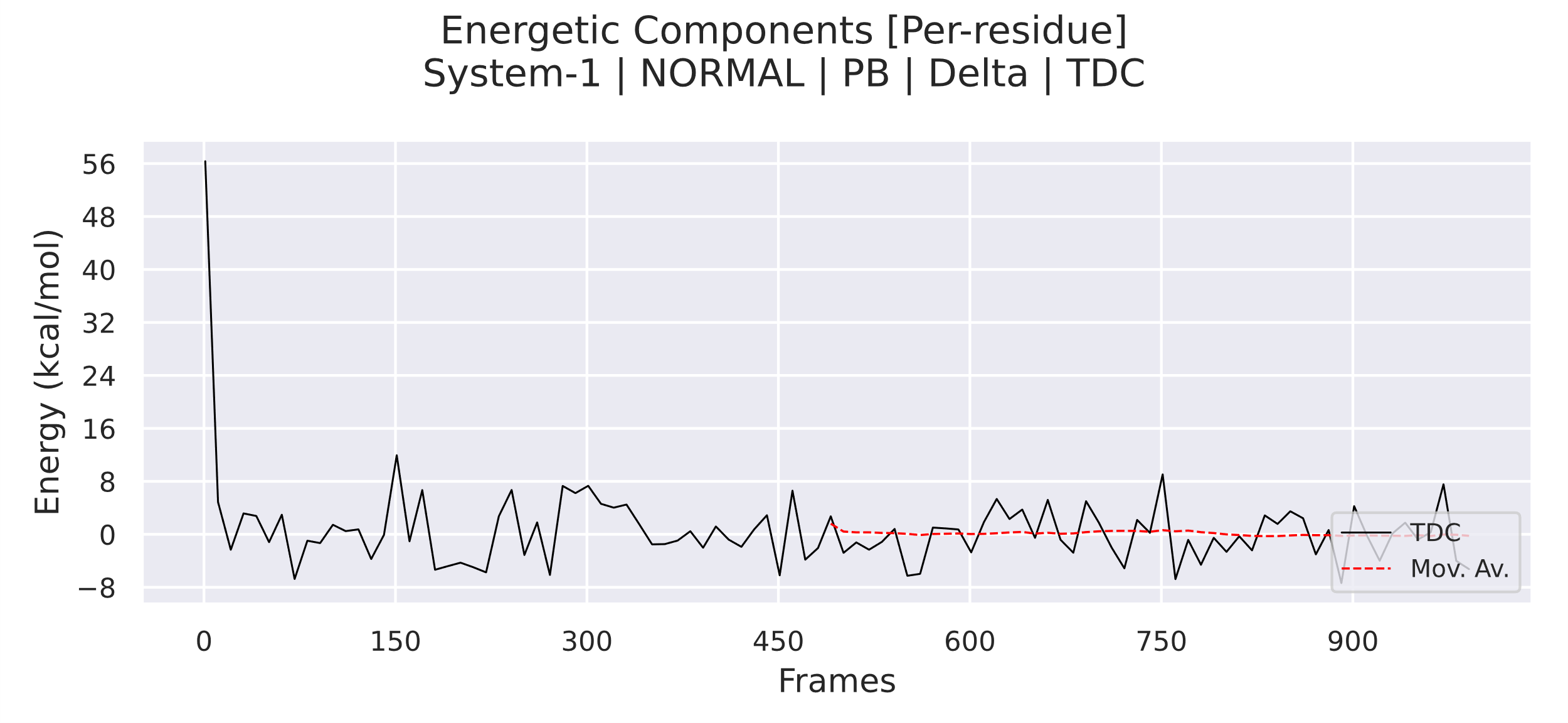 | 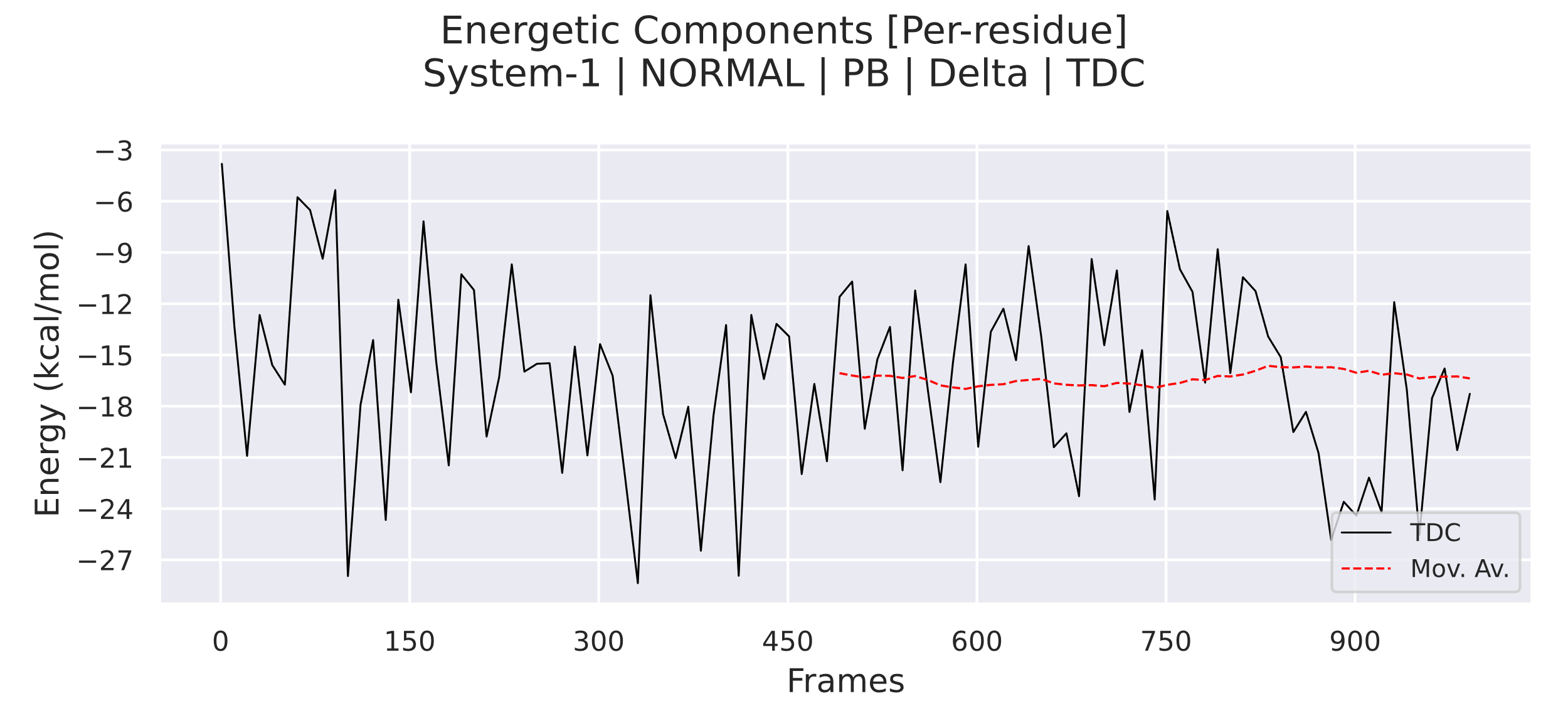 |

**Figure S10.** MM-PBSA binding free energy decomposition of α-glucosidase–ligand complexes. Component energy terms for acarbose (VDWAALS, EEL, EPB, TOTAL) (b, d, f, h). Corresponding component energy terms for berberine (a, c, e, g).

**Pharmacokinetics and Toxicity Prediction**

**Pharmacokinetic and toxicity predictions indicated that berberine is predicted to possess** favorable oral drug-like properties, with moderate lipophilicity (MLOGP ≈ 2.2), high gastrointestinal absorption, and compliance with Lipinski’s rule of five. It was also predicted to cross the blood–brain barrier and act as a substrate of P-glycoprotein, while inhibiting multiple CYP isoforms (CYP1A2, CYP2D6, and CYP3A4), suggesting potential drug–drug interactions. Toxicity analysis classified berberine in toxicity class 3 with an estimated LD₅₀ of 200 mg·kg⁻¹, **and generated in silico alerts for possible neurotoxic, immunotoxic, and carcinogenic effects, which have not been experimentally confirmed**, despite the absence of predicted hepatotoxicity and nephrotoxicity. **Overall, the in silico data suggest that while berberine is predicted to demonstrate** favorable pharmacokinetic behavior, its predicted safety concerns necessitate further experimental validation (**Table S5** and **Table S6**)**.** Pharmacokinetic properties of berberine predicted by SwissADME.

| **Pharmacokinetics** | | |
| --- | --- | --- |
| **Parameters** | | **BBR** |
| ***Physicochemical properties*** | Molecular mass (g/mol) | 336.36 g/mol |
|  | Num. heavy atoms | 25 |
|  | Num. arom. heavy atoms | 16 |
|  | Num. rotatable bonds | 2 |
|  | Num. H-bond acceptors | 4 |
|  | Num. H-bond donors | 0 |
|  | Molar Refractivity | 94.87 |
|  | TPSA | 40.80 Å² |
| ***Lipophilicity*** | Log Po/w (MLOGP) | 2.19 |
| ***Water Solubility*** | Solubility class | Moderately soluble |
|  | Log s (ESOL) | -4.55 |
| ***Pharmacokinetics*** | GI absorption | High |
|  | BBB permeant | Yes |
|  | P-gp substrate | Yes |
|  | CYP1A2 inhibitor | Yes |
|  | CYP2C19 inhibitor | No |
|  | CYP2C9 inhibitor | No |
|  | CYP2D6 inhibitor | Yes |
|  | CYP3A4 inhibitor | Yes |
| ***Drug-likeness*** | Lipinski | Yes; 0 violation |
|  | Bioavailability score | 0.55 |
| GI: Gastrointestinal; CNS: Central nervous system; BBB; Blood-Brain Barrier; P-gp: P-glycoprotein | | |

**Table S6.** Predicted toxicity profile of berberine from ProTox-III.

| **Toxicity** | |
| --- | --- |
| **Parameters** | **BBR** |
| Predicted LD50 | 200 mg/kg |
| Predicted Toxicity Class | 3 |
| Hepatotoxicity | Inactive |
| Neurotoxicity | Active |
| Nephrotoxicity | Inactive |
| Respiratory toxicity | Active |
| Cardiotoxicity | Inactive |
| Carcinogenicity | Active |
| Immunotoxicity | Active |
