# Supplementary material for: Probiotics, synbiotics and berberine in Type 2 diabetes mellitus: A systematic review, meta-analysis, and molecular dynamics simulation study
Source: PLoS One. 2026 May 29;21(5):e0348907. doi: 10.1371/journal.pone.0348907 (PMC13221027; doi:10.1371/journal.pone.0348907)
Supplement: Attachment — Submitted filename: renamed_bc754.docx [file pone.0348907.s004.docx]

**Reviewer’s Comment**

1. The integration of the clinical meta-analysis with the in-silico component is conceptually misaligned. The molecular docking and molecular dynamics analyses focus exclusively on α-glucosidase inhibition, which does not represent the primary biological mechanisms through which probiotics influence glycemic control.
2. Key mechanisms attributed to berberine, including AMPK activation, modulation of gut microbiota, and bile acid signaling, are not captured by the selected computational target.
3. Consequently, the in-silico analyses do not adequately explain the pooled clinical outcomes, particularly for probiotic interventions, and may give the impression of mechanistic validation that is not fully supported. The authors should either clearly reframe the in-silico work as exploratory and berberine-specific or remove claims suggesting consistency between the computational findings and the overall clinical results.
4. The pooled meta-analysis combines interventions with substantial biological and clinical heterogeneity, including single-strain probiotics, multi-strain probiotics, synbiotics, berberine monotherapy, combination therapies, and diet or fecal microbiota transplantation comparators. These interventions differ fundamentally in mechanism of action and clinical context. The very high heterogeneity observed for both fasting plasma glucose and HbA1c (I² > 85%) indicates that the pooled estimates may represent averages that are difficult to interpret clinically.
5. Separate analyses for probiotics/synbiotics, berberine alone, and combination therapies are warranted to improve interpretability and validity.
6. The clinical significance of the findings appears overstated. The pooled reduction in HbA1c (approximately −0.19%) is below commonly accepted thresholds for clinically meaningful change. Although this limitation is briefly acknowledged, it is not sufficiently emphasized in the interpretation.
7. Statements implying clinically meaningful improvement, particularly for fasting plasma glucose, should be tempered. The results are more consistent with modest, adjunctive effects rather than effects likely to influence standard treatment decisions.
8. Risk of bias and publication bias require more thorough exploration. Funnel plots for both primary outcomes show asymmetry, suggesting potential small-study effects, yet Egger’s test results are not quantitatively reported. Several included trials are small, short in duration, and likely underpowered, which may inflate effect estimates. These issues should be explicitly addressed, and Egger’s test p-values reported, along with a clearer discussion of the implications for the robustness of the findings.
9. The in-silico section is disproportionately long relative to its contribution to the study’s conclusions. Extensive reporting of molecular dynamics, principal component analysis, free energy landscapes, and MM-PBSA calculations exceeds what is necessary to support the main claims.
10. The manuscript would benefit from language editing to address run-on sentences, repetition, and inconsistent tense usage.
11. Table 2 is overly dense and difficult to interpret and should be simplified or divided for clarity.
12. Some references are dated or repeated without clear differentiation.
13. The ethics statement marked as “N/A” is acceptable but should explicitly state that the study is a secondary analysis of previously published data.

**Recommendation** :::::: Major revision.
