# Supplementary material for: Probiotics, synbiotics and berberine in Type 2 diabetes mellitus: A systematic review, meta-analysis, and molecular dynamics simulation study
Source: PLoS One. 2026 May 29;21(5):e0348907. doi: 10.1371/journal.pone.0348907 (PMC13221027; doi:10.1371/journal.pone.0348907)
Supplement: Attachment — Submitted filename: Response to Reviewers.docx [file pone.0348907.s006.docx]

**Response to Reviewers**

Manuscript ID: PONE-D-25-68086
Title: Probiotics, synbiotics and Berberine in Type 2 Diabetes Mellitus: A Systematic Review, Meta-Analysis, and Molecular Dynamics Simulation Study

“All revisions have been highlighted in red in the Revised Manuscript with Track Changes.”

**Response to the Academic Editor**

**Editor Comment 1:**
The authors need to justify the significance of the study, relate it to the existing literature, and clearly identify the gap in current knowledge that this study addresses.

**Author response:** We thank the Academic Editor for this comment. The Introduction and Discussion sections have been revised to more clearly articulate the significance of this study and its relationship to existing literature. Specifically, we now emphasize that although probiotics, synbiotics, and berberine have been widely investigated as adjunctive therapies for type 2 diabetes mellitus, previous meta-analyses have typically evaluated these interventions separately and without integration of mechanistic in silico analyses. The key knowledge gap addressed by our study is the lack of a comprehensive synthesis that combines clinical evidence with exploratory molecular-level insights for berberine while simultaneously assessing probiotics and synbiotics in a unified framework. These revisions clarify how our work extends prior studies by providing updated pooled estimates, subgroup analyses (probiotics vs placebo), and an integrated clinical computational perspective.

**Editor Comment 2:**
The limitations of the study should be clearly discussed.

**Author response:** We agree and have expanded the Discussion section to explicitly address the main limitations of the study. These include the high between-study heterogeneity, small sample sizes and short durations of many included trials, variability in probiotic strains and dosing regimens, and the potential influence of publication bias and small-study effects. We also acknowledge methodological limitations such as incomplete reporting of randomization and selective outcome reporting in some trials. In addition, we clarify that the in silico analyses model only a single molecular target and cannot capture systemic or microbiota-mediated mechanisms. These limitations are now discussed in greater depth to ensure transparent interpretation of the findings.

**Editor Comment 3:**
The authors should provide recommendations for future research.

**Author response:** We have added explicit recommendations for future research in the Discussion section. We now suggest that future randomized controlled trials should be larger, longer in duration (≥24 weeks), and standardized with respect to probiotic strain composition and berberine dosing. We further recommend that future studies incorporate mechanistic endpoints (e.g., microbiota profiling, inflammatory markers) and comprehensive safety assessments, including drug drug interaction monitoring. These additions clarify how subsequent research can address current uncertainties and strengthen clinical applicability.

**Editor Comment 4:**
Serious concerns raised by the reviewers affecting pivotal sections of the study must be addressed critically before resubmission.

**Author response:** We have carefully addressed all substantive concerns raised by the reviewers, particularly those related to risk of bias, publication bias, interpretation of computational findings, safety reporting, and overstatement of clinical relevance. Revisions were made to the Methods, Results, and Discussion sections to report Egger’s test p-values, clarify that computational findings are exploratory and predictive, acknowledge short trial durations and underpowered studies, and align conclusions with the low-to-moderate certainty of evidence based on GRADE. These changes ensure that the pivotal sections of the manuscript now reflect a cautious, transparent, and methodologically rigorous interpretation of the data.

**Response to the Reviewer #1**

**Reviewer #1:** This manuscript addresses an important and timely topic: the potential role of probiotics, synbiotics, and berberine as adjunctive interventions in the management of type 2 diabetes mellitus. The inclusion of a large number of randomized controlled trials, adherence to PRISMA guidelines, PROSPERO registration, and the use of GRADE to assess certainty of evidence are strengths of the study. The topic is relevant to both clinical and translational research audiences, and the authors have made a substantial effort to synthesize a broad and complex literature.

However, several major issues limit the interpretability and robustness of the findings and should be addressed before the manuscript can be considered for publication.

**Author response:** We thank the reviewer for their thoughtful and constructive evaluation of our manuscript and for highlighting both its strengths and important areas for improvement. We have carefully revised the manuscript in response to all major and minor comments, as detailed below.

**1.** First, there is a conceptual mismatch between the clinical meta-analysis and the in-silico component of the study. The molecular docking and molecular dynamics analyses focus exclusively on α-glucosidase inhibition, which does not reflect the dominant biological mechanisms by which probiotics influence glycemic control. In addition, key mechanisms attributed to berberine, including AMPK activation, modulation of gut microbiota, and bile acid signaling, are not captured by the chosen computational target. As a result, the computational analyses do not adequately explain the pooled clinical outcomes, particularly for probiotic interventions, and risk giving an impression of mechanistic validation that is not fully supported. The authors should either clearly reframe the in-silico work as exploratory and berberine-specific or remove strong claims suggesting consistency between the computational findings and the overall clinical results.

**Author response:** Regarding heterogeneity, we acknowledge that the included interventions differ biologically and clinically. We have revised the Results and Discussion sections to clarify that pooled estimates represent average adjunctive effects across heterogeneous interventions. Where data permitted, we conducted additional subgroup analyses separating probiotics/synbiotics, berberine monotherapy, and combination therapies, and we now interpret pooled findings with greater caution. The high I² values are explicitly reported and discussed as a limitation affecting clinical interpretability.

2. Second, the meta-analysis pools interventions with substantial biological and clinical heterogeneity. The included studies encompass single-strain probiotics, multi-strain probiotics, synbiotics, berberine monotherapy, combination therapies, and diet or fecal microbiota transplantation comparators. These interventions differ fundamentally in mechanisms of action and clinical context. The very high heterogeneity observed for both fasting plasma glucose and HbA1c (I² > 85%) suggests that the pooled estimates may represent clinically uninterpretable averages. Subgroup analysis based only on treatment duration is insufficient to address this issue. Separate meta-analyses for probiotics/synbiotics alone, berberine alone, and combination therapies (where data permit) would substantially improve interpretability and analytical rigor.

**Author response:** Thank you for your valuable comments. Regarding heterogeneity, we acknowledge that the included interventions differ biologically and clinically. We have revised the Results and Discussion sections to clarify that pooled estimates represent average adjunctive effects across heterogeneous interventions. Where data permitted, we conducted additional subgroup analyses separating probiotics/synbiotics, berberine monotherapy, and combination therapies, and we now interpret pooled findings with greater caution. The high I² values are explicitly reported and discussed as a limitation affecting clinical interpretability.

3. Third, the clinical significance of the findings appears overstated. The pooled reduction in HbA1c of approximately −0.19% is below commonly accepted thresholds for clinically meaningful change. While this limitation is briefly acknowledged, it is not sufficiently emphasized in the interpretation. Statements implying clinically meaningful improvement, particularly in relation to fasting plasma glucose, should be tempered. The results are more consistent with modest, adjunctive effects rather than effects likely to influence standard therapeutic decision-making.

**Author response:** We agree that the clinical significance of the observed HbA1c reduction is modest. We have revised the Results, Discussion, and Conclusion to avoid overstating clinical relevance and now describe the observed effects as small but statistically significant adjunctive improvements rather than clinically decisive therapeutic effects.

4. Fourth, risk of bias and publication bias are not explored in sufficient depth. Funnel plot asymmetry is visible for both primary outcomes, suggesting potential small-study effects, yet Egger’s regression results are not quantitatively reported. Many included trials are small, short in duration, and likely underpowered, which may inflate effect sizes and contribute to heterogeneity. These issues should be explicitly addressed, with reporting of Egger’s test p-values and a clearer discussion of how potential publication bias and small-study effects may influence the conclusions.

**Author response:** Risk of bias and publication bias assessments have been expanded in the revised manuscript. Egger’s regression test p-values are now explicitly reported for both primary outcomes and for the probiotics-only HbA1c subgroup analysis. In addition, the Discussion section has been revised to provide a more detailed evaluation of potential small-study effects, short intervention durations, and underpowered trials as contributors to heterogeneity and possible inflation of effect estimates.

Fifth, the in-silico section is disproportionately long relative to its contribution to the overall conclusions. Detailed reporting of molecular dynamics simulations, principal component analysis, free energy landscapes, and MM-PBSA calculations exceeds what is necessary to support the study’s claims. Given PLOS ONE’s emphasis on methodological soundness rather than mechanistic novelty, the computational results should be substantially condensed, with extended analyses and figures moved to the Supplementary Information.

**Author response:** The in silico section has been substantially condensed, with detailed molecular dynamics analyses, free energy landscapes, and MM-PBSA data moved to the Supplementary Information. The main text now reports only essential findings needed to support the exploratory computational objective, consistent with the journal’s emphasis on methodological soundness.

There are also several minor issues that require attention. The manuscript would benefit from language editing to address run-on sentences, repetition, and inconsistent tense usage. Table 2 is extremely dense and difficult to interpret and should be simplified or divided to improve readability. Some references are dated or repeated without sufficient clarification. The ethics statement marked as “N/A” is acceptable; however, it should explicitly state that the study is a secondary analysis of previously published data.

Overall, these revisions improve conceptual alignment, interpretability, and transparency, and better reflect the modest and adjunctive nature of the observed clinical effects.

In summary, while the study addresses a relevant question and is methodologically grounded in several respects, the issues outlined above limit confidence in the conclusions. Addressing the conceptual alignment between clinical and computational components, appropriately handling heterogeneity, tempering claims of clinical significance, and strengthening bias assessment are necessary for the manuscript to meet the standards expected for publication.

**Author response:** Minor issues were also addressed. The manuscript was edited for language clarity and tense consistency. Table 2 was simplified for improved readability. Redundant and outdated references were corrected. The ethics statement now explicitly states that this study is a secondary analysis of previously published data.

**Response to the Reviewer #2**

**Reviewer #2:** This manuscript presents a well-structured systematic review and meta-analysis of probiotics and synbiotics in type 2 diabetes, complemented by in silico mechanistic analyses of berberine against α-glucosidase. The methodology is generally sound, including appropriate meta-analytic techniques, heterogeneity assessment, and risk-of-bias evaluation, and the integration of computational modeling adds conceptual depth. However, several conclusions particularly regarding clinical relevance and mechanistic implications are occasionally overstated and should be tempered to reflect the modest effect sizes and substantial heterogeneity observed. With careful revision the manuscript would make a valuable contribution to the field.

**Author response:** We thank the reviewer for the constructive and detailed comments, which have helped improve clarity, accuracy, and tone of the Introduction. We have revised the Introduction to (i) strengthen claims with appropriate citations, (ii) correct formatting and grammatical issues, (iii) soften overly strong or speculative statements, (iv) reduce redundancy, and (v) reframe novelty and mechanistic claims in a more cautious and balanced manner consistent with PLOS ONE standards. All requested changes have been implemented as outlined below.

***Introduction***

1. “Type 2 diabetes mellitus (T2DM) is one of the most pressing public health challenges worldwide.”

This statement is clear but somewhat generic. Add a supporting citation directly within this sentence to strengthen its impact and align with PLOS ONE’s preference for evidence-supported claims.

**Author response:** A citation has been added to support this statement.

2.“The disease is associated with substantial morbidity and mortality, primarily due to its long-term complications such as cardiovascular disease, nephropathy, neuropathy, and retinopathy (2007).”

The citation is incomplete and does not follow proper referencing format. Replace “(2007)” with a complete and correctly formatted citation according to PLOS ONE guidelines.

**Author response:** The incomplete citation has been replaced with a properly formatted reference.

3.“The pathophysiology of T2DM is multifactorial, characterized by insulin resistance in peripheral tissues, progressive pancreatic β-cell dysfunction, and chronic hyperglycemia (Galicia-Garcia et al., 2020).”

There is a spacing error before the citation. Insert a space between “hyperglycemia” and the citation. Additionally, ensure the citation format complies with PLOS ONE numbered style.

**Author response:** The spacing error has been corrected and citation formatting standardized.

4.“Dysbiosis an imbalance in the composition and function of intestinal microbiota has been linked to systemic inflammation…”

A comma is required after “Dysbiosis.” Revise to: “Dysbiosis, an imbalance in the composition and function of intestinal microbiota, has been linked…”

**Author response:** Corrected.

5.“Furthermore, none of the current standard treatments directly address the underlying disturbances in gut microbiota or the metabolic inflammation associated with T2DM (Caturano et al., 2024).”

This statement may be overly strong, as some treatments (e.g., metformin) have documented microbiota effects. Consider softening the claim to: “Most current standard treatments do not primarily target disturbances in gut microbiota…” and ensure appropriate citation support.

**Author response:** The statement has been softened to reflect that most, but not all, treatments primarily target gut microbiota.

6. “Recent advances in microbiome research have emphasized the pivotal role of gut microbiota in the development and progression of T2DM.”

This sentence overlaps conceptually with earlier microbiota discussion. Consider merging this paragraph with the earlier microbiota section to reduce redundancy and improve flow.

**Author response:**  The overlapping sentences were merged to improve flow and avoid repetition.
7. “Among the emerging therapeutic compounds that target both metabolic pathways and gut microbiota, BBR has shown particular promise.”

The abbreviation “BBR” is used before being defined. Revise to: “Berberine (BBR) has shown particular promise…” at first mention.

**Author response:**  Berberine (BBR) is now defined at first mention.

8.“In addition, BBR has been shown to beneficially modulate gut microbiota, suggesting a shared mechanistic pathway with probiotics (Huang et al., 2022; Wang et al., 2022; Wang et al., 2022; Yang et al., 2023).”

The citation “Wang et al., 2022” appears twice. Remove the duplicate reference and verify citation accuracy.

**Author response:**  Duplicate reference removed.

9.“This overlap raises the possibility of synergistic effects when probiotics and berberine are used in combination.”

This statement is speculative. Consider softening it to: “This overlap suggests potential complementary or synergistic mechanisms that warrant further investigation.”

**Author response:** Revised to a more cautious formulation indicating that synergy warrants further investigation.

10.“Critically, no previous systematic review has comprehensively evaluated the combined effects of probiotics and berberine…”

This is a strong novelty claim and may be difficult to substantiate. Revise to: “To our knowledge, no previous systematic review has comprehensively evaluated…” or “Few studies have systematically evaluated…” unless a documented search strategy confirms the claim.

**Author response:**  Reworded using “to our knowledge” and “few studies” to avoid overstatement.

11.“By combining evidence from both clinical trials and in silico analyses, our study seeks to provide robust and mechanistically informed insights…”

The wording is slightly promotional. Consider using more neutral language consistent with PLOS ONE standards, such as: “This integrated approach aims to provide complementary clinical and mechanistic evidence…”

**Author response:**  Replaced with neutral phrasing consistent with PLOS ONE style.

12.“Given these gaps in the current literature, a rigorous synthesis of available evidence is warranted.”

This sentence is appropriate but could be strengthened by briefly specifying the identified gap (e.g., heterogeneity, lack of combined evaluation, absence of mechanistic validation) to improve precision.

**Author response:**  Revised to explicitly specify the gap (heterogeneity, lack of integrated evaluation, and limited mechanistic synthesis).

***Method***

2.1. Search strategy and study selection

1.“A systematic literature search was conducted in PubMed, Scopus, Cochrane Library, Web of Science, and Embase from 2015 to 2025…”

The search timeframe restriction (2015–2025) requires justification. Briefly explain why studies published before 2015 were excluded to reduce concerns about potential selection bias.

**Author response:**  Justification added to explain focus on contemporary interventions and diagnostic criteria.

2. “Two authors were initially listed at registration; additional authors contributed during the review and are included in this publication.”

This statement raises authorship transparency concerns. Clarify whether the PROSPERO record was formally updated and confirm that all listed authors meet established authorship criteria.

**Author response:**  We have revised the Methods section to clarify authorship contribution. The manuscript now states that additional contributors participated in data extraction, analysis, and manuscript preparation and are included as co-authors in accordance with standard authorship criteria.

2.3. Data synthesis and analysis

1.“A random-effects model was applied to account for between-study variability.”

This statement is appropriate but incomplete. Specify which estimator was used (e.g., DerSimonian–Laird, REML) to improve methodological transparency.

**Author response:**  Restricted maximum likelihood (REML) specified.

2.“Publication bias was evaluated using funnel plot inspection and Egger’s test.”

Clarify whether Egger’s test was performed only when at least 10 studies were available, as recommended in meta-analysis guidelines.

**Author response:**  Clarified that Egger’s test was applied only when ≥10 studies were available.

3.“All statistical analyses and plots were conducted using R software.”

This statement lacks reproducibility detail. Specify the R version and the packages used (e.g., meta, metafor) to enhance transparency.

**Author response:**  R version and packages specified.

2.4. The quality of evidence

“Summary of Findings (SoF) tables were generated…”

Indicate whether GRADEpro software or another tool was used to construct the SoF tables, as this improves reproducibility.

**Author response:**  GRADEpro GDT software specified.

2.5.3. Molecular docking

1.“Default exhaustiveness parameters were applied…”

The term “default” is insufficient for reproducibility. Specify the exact exhaustiveness value used in AutoDock Vina.

**Author response:**  We have revised the manuscript to report the exact exhaustiveness parameter used in AutoDock Vina (exhaustiveness = 8) to improve reproducibility.

2. “The best binding pose of berberine was selected based on the lowest binding energy…”

Selecting only the lowest energy pose may introduce bias. Clarify whether clustering analysis or consistency across multiple docking runs was considered.

**Author response:**  We have clarified that docking was repeated multiple times and that binding poses were evaluated based on consistency of binding orientation and clustering, in addition to lowest binding energy.

2.5.4. Molecular dynamics simulation

1.“…with default settings…”

The phrase “default settings” should be avoided. All simulation parameters appear listed; therefore, remove this phrase to avoid ambiguity.

**Author response:**  We have removed the phrase “default settings” and now describe all simulation parameters explicitly to avoid ambiguity.

2.“Production simulations were run for 100 ns…”

Provide justification for selecting a 100 ns simulation time and explain whether convergence was assessed.

**Author response:**  We have added justification that a 100 ns simulation length was selected based on prior literature and that trajectory convergence was assessed using RMSD stabilization.

3.“…in-house Python scripts.”

Specify whether these scripts are publicly available (e.g., GitHub or supplementary material) to enhance transparency and reproducibility.

**Author response:**  We have clarified that the Python scripts used for trajectory analysis are publicly available in the GitHub repository associated with this study.

***3. RESULTS***

3.1. Literature search

1.“A systematic search of PubMed, Scopus, Cochrane Library, Web of Science and Embase was performed according to the predefined protocol…”

This sentence is appropriate but largely repeats information from the Methods section. Consider shortening it to avoid redundancy and focus the Results section on numerical outcomes of the search (e.g., number of records identified, screened, excluded).

**Author response:** We have shortened this sentence to focus on numerical search outcomes and avoided repeating methodological details already described in the Methods section.

2. “After eligibility assessment, the randomized controlled trials included in the quantitative synthesis comprised the following key studies: Perraudeau et al., 2020; Zhang et al., 2020; …”

Listing all included studies in the main text reduces readability and interrupts narrative flow. Replace this list with a summary statement (e.g., “A total of 32 RCTs were included”) and refer readers to Table 2 for detailed study information.

**Author response:** We replaced the list of individual studies with a summary statement indicating the total number of included RCTs and directed readers to Table 2 for detailed study characteristics.

3.2. Description of included studies

1.“Sample sizes varied considerably, ranging from fewer than 20 participants in pilot studies to more than 200 in large multi-center trials.”

Provide the exact minimum and maximum sample sizes to enhance precision and transparency.

**Author response:** We have revised the text to report the exact minimum and maximum sample sizes. [Sample sizes varied widely, with total study enrollment ranging from 13 participants (8 intervention and 5 control) [50] to 213 participants (103 intervention and 110 control)]

2.“Despite methodological differences… the included studies provided a comprehensive dataset…”

The term “comprehensive dataset” is somewhat interpretative. Consider replacing it with neutral wording such as “provided sufficient data for quantitative synthesis.”

**Author response:** We replaced this phrase with neutral wording indicating that the studies provided sufficient data for quantitative synthesis.

3.“Across the body of evidence, certain patterns emerged.”

This phrase is vague. Replace it with a more specific statement summarizing the observed trend.

**Author response:** We replaced this phrase with a more specific description of observed trends across interventions and publication periods.

4.“More recent studies (2020–2025) increasingly focused on probiotics…”

This statement requires quantitative support. Indicate the number or proportion of studies published during this period to substantiate the claim.

**Author response:** We now report the number of studies published during 2020–2025 to substantiate this statement. Which is 16 in number; 50%.

3.5.2. MD Simulation Results

1.“Four key structural parameters root mean square deviation (RMSD), root mean square fluctuation (RMSF), radius of gyration (Rg), and solvent-accessible surface area (SASA), along with hydrogen bond analysis were investigated…”

A comma is required after “parameters.” Revise to: “Four key structural parameters, root mean square deviation (RMSD), root mean square fluctuation (RMSF), radius of gyration (Rg), and solvent-accessible surface area (SASA), along with hydrogen bond analysis, were investigated…”

**Author response:** The sentence has been revised to include the required comma after “parameters” for grammatical correctness.

2.“The RMSD profiles revealed that all systems reached equilibrium after the initial 10 ns…”

This claim requires quantitative support. Provide the average RMSD values and standard deviations for each system to substantiate the assertion of equilibrium.

“Importantly, the test compound maintained a relatively stable RMSD…”

The word “Importantly” is interpretative. Remove this term and present the observation in neutral language.

**Author response:** The statement regarding equilibration has been revised to include average RMSD values with standard deviations for each system, and the interpretative term “Importantly” has been removed to maintain neutral reporting.

3.“These results are consistent with RMSD and hydrogen bond analyses…”

This statement is interpretative. Instead, explicitly state the numerical findings rather than drawing qualitative reinforcement.

**Author response:** The sentence has been revised to explicitly describe the numerical trends observed in the PCA and hydrogen bond analyses rather than using qualitative reinforcement language.

4.“Collectively, these findings demonstrate that both the standard and test compound induce structural stabilization…”

The word “demonstrate” may overstate the evidence. Replace with “suggest” to maintain cautious scientific interpretation.

**Author response:** The wording has been softened by replacing “demonstrate” with “suggest” to avoid overstatement of computational findings.

3.5.3. MM-PBSA Binding Free Energy Analysis

1.“The results demonstrated that the standard ligand (acarbose) exhibited a substantially more favorable binding free energy…”

Replace “demonstrated” with “indicated” or “suggested” to avoid overstating computational predictions.

**Author response:** The term “demonstrated” has been replaced with “indicated” to reflect the predictive nature of MM-PBSA calculations.

2.“These values emphasize that the favorable balance… underlies its stronger binding affinity.”

This is mechanistically interpretative. Consider softening to: “These values suggest that the balance of van der Waals and non-polar interactions contributes to the stronger binding affinity.”

**Author response:** The sentence has been revised to state that the energy components “suggest” rather than “emphasize” mechanistic contributions.

3.“Overall, the MM-PBSA results support the dynamic analyses…”

Replace “support” with “are consistent with” to maintain neutrality.

**Author response:** The wording has been revised to maintain neutral interpretation of computational results.

3.5.4. Pharmacokinetics and Toxicity Prediction

1.“Pharmacokinetic and toxicity predictions indicated that berberine possesses favorable oral drug-like properties…”

Since these are computational predictions, replace “possesses” with “is predicted to possess” to avoid implying experimental validation.

**Author response:** We thank the reviewer for this suggestion. The wording has been revised to reflect the predictive nature of the computational analysis by replacing “possesses” with “is predicted to possess.”

2.“…highlighting possible neurotoxic, immunotoxic, and carcinogenic risks…”

This statement may appear alarming without context. Clarify that these are in silico toxicity alerts and not experimentally confirmed toxicities.

**Author response:** We agree with the reviewer. The text has been revised to clarify that the reported neurotoxic, immunotoxic, and carcinogenic risks are based on in silico toxicity alerts and are not experimentally confirmed.

3.“Overall, the in silico data suggest that while berberine demonstrates good pharmacokinetic behavior…”

Replace “demonstrates” with “is predicted to demonstrate” for accuracy.

**Author response:** The wording has been revised accordingly to emphasize that these findings are derived from computational predictions rather than experimental validation.

3.6. Safety and Tolerability

1.“Probiotics and synbiotics were consistently well tolerated, with no serious adverse events reported…”

Clarify whether adverse event reporting was complete across all trials, as underreporting may bias safety conclusions.

**Author response:** We have revised the safety section to acknowledge that adverse event reporting may not have been complete across all trials and that underreporting cannot be excluded.

2.“Taken together, probiotics appear safe and well tolerated…”

This statement is acceptable but should acknowledge the duration of follow-up (mostly short-term trials) to avoid overgeneralization.

**Author response:** We have modified the text to explicitly note that safety conclusions are based largely on short-term trials and should be interpreted cautiously.

***4. Discussion***

1.“These clinical results should, however, be interpreted in the context of substantial between-study heterogeneity…”

This is appropriate; however, specify the I² values here for clarity rather than relying on earlier sections.

**Author response:** We have revised the Discussion to explicitly report the degree of heterogeneity for both primary outcomes (I² > 85%) to improve clarity and transparency.

2.“The FPG decrease… is likely clinically relevant…”

The phrase “likely clinically relevant” requires justification. Provide a reference or predefined clinical benchmark.

**Author response:** We have revised the text to contextualize the fasting plasma glucose reduction using commonly applied clinical benchmarks (≈0.5–1.0 mmol·L⁻¹) and to clarify that the observed effect lies at the lower end of this range.

3.“These computational results are consistent with the clinical picture…”

This may imply causality between computational binding and clinical outcomes. Clarify that the mechanistic link is hypothetical and indirect.

**Author response:** We have revised the wording to clarify that the relationship between in silico binding results and clinical outcomes is indirect and hypothetical, and that the computational findings are exploratory rather than mechanistic validation.

4.“The results support the potential role of probiotics/synbiotics and berberine as adjunctive interventions…”

This conclusion is acceptable but should explicitly state that certainty of evidence was low to moderate per GRADE.

**Author response:** We have revised the Discussion to explicitly note that the certainty of evidence for the primary outcomes was low to moderate according to GRADE.

***6. Conclusion***

1.“Our integrated meta-analysis and in silico work indicate modest benefits…”

Replace “indicate” with “suggest” to maintain cautious interpretation.

**Author response:** we have replaced “indicate” with “suggest”

2.“Computational data show berberine binds α-glucosidase less stably than acarbose…”

Replace “show” with “suggest” or “predict” because binding stability was derived from simulations.

**Author response:** we have replaced “show” with “suggest”

3.“Given the small HbA1c effect, these agents may be considered adjuncts…”

This recommendation is appropriate but should emphasize that clinical decisions must be individualized and guided by clinical guidelines.

**Author response:** We thank the reviewer for this valuable suggestion. We have revised the Conclusion to emphasize that probiotics, synbiotics, and berberine should be considered only as adjunctive strategies and that any clinical use should be based on individualized patient assessment and in accordance with established clinical guidelines. This change ensures that our conclusions do not imply generalized clinical recommendations and remain aligned with current standards of care.

***7.Reference***

Your submission does not fully follow PLOS ONE formatting requirements. References should be listed at the end of the manuscript and numbered in the order that they appear in the text. In the text, cite the reference number in square brackets (e.g., “We used the techniques developed by our colleagues [19] to analyze the data”). PLOS uses the numbered citation (citation-sequence) method and first six authors, et al..

**Author response:** Thank you for your valuable comments. We have carefully revised the references to fully comply with the PLOS ONE formatting guidelines. References are now listed in numerical order according to their appearance in the text, and citations are presented in square brackets following the citation-sequence method. We have formatted the reference list in Vancouver style, including the first six authors followed by et al. where applicable.

**Response to the Reviewer #3**

**Reviewer #3:** Thank you for the opportunity to review this manuscript. The idea of using a multi-objective optimization framework to personalize insulin infusion using CGM and insulin delivery data is clinically relevant, and the inclusion of safety constraints and subject-specific modeling is appropriate.

**Author response:** We respectfully note that Reviewer #3’s comments appear to refer to a different manuscript involving insulin infusion optimization and CGM-based modeling. Our study is a systematic review and meta-analysis of probiotics, synbiotics, and berberine for glycemic control and does not involve insulin delivery, control algorithms, or predictive modeling. We therefore believe these comments may have been inadvertently assigned to our manuscript.

That said, a few areas need clarification before the results can be fully assessed. First, the objectives and the way the optimization problem is set up are not always clearly aligned (for example, it is not consistently clear whether time-in-range is directly optimized or inferred from other metrics). Second, the Results sometimes shift between detailed reporting on three representative subjects and broader cohort-level summaries; I recommend making this distinction explicit throughout the text, figures, and tables so the reader can tell what is subject-level versus group-level evidence.

**Author response:**

More detail is also needed on the prediction model used for offline evaluation, including how it was trained and validated, what its typical error looks like, and how model error might affect the safety constraints and the reported improvements. Lastly, the statistical comparisons would benefit from clearer description of the analysis unit (what counts as a paired observation, and what sample is being compared). I also noticed some introduction statements about multimodal/imaging integration that do not appear elsewhere in the methods or results; please revise those sections so they match what was actually done.

**Author response:**

Addressing these items would improve transparency, reproducibility, and interpretability of the findings.

**Author response:**
