# Supplementary material for: Probiotics, synbiotics and berberine in Type 2 diabetes mellitus: A systematic review, meta-analysis, and molecular dynamics simulation study
Source: PLoS One. 2026 May 29;21(5):e0348907. doi: 10.1371/journal.pone.0348907 (PMC13221027; doi:10.1371/journal.pone.0348907)
Supplement: Attachment — Submitted filename: Response_to_Reviewers_auresp_2.docx [file pone.0348907.s007.docx]

**Response to Reviewers**

We sincerely thank the Editor and the Reviewers for their careful evaluation of our manuscript and for the constructive comments that helped improve the clarity, rigor, and interpretation of the study. We have carefully revised the manuscript in accordance with the reviewers’ suggestions. All changes made in the revised manuscript are highlighted using red text for ease of identification.

Below we provide a point-by-point response to the reviewers’ comments.

**Reviewer #2**

**Comment:**
The authors have addressed most of the comments and substantially improved the clarity, methodological transparency, and balance of interpretation throughout the manuscript. In particular, the revised version appropriately moderates claims regarding clinical relevance, clarifies the exploratory nature of the in silico analyses, and provides additional methodological details for the meta-analysis and molecular simulations. The discussion now better contextualizes the modest effect sizes and acknowledges the substantial heterogeneity across trials, which strengthens the scientific rigor of the manuscript. While a few minor editorial issues remain, these do not affect the overall quality or interpretation of the work. For example, small grammatical corrections may still be required, such as adding a comma in “Dysbiosis an imbalance in the composition and function of intestinal microbiota…”, which should read “Dysbiosis, an imbalance in the composition and function of intestinal microbiota…”, and correcting minor wording such as “50% of study” to “50% of studies”. Some sentences could also be clarified for greater precision. For instance, “The RMSD profiles revealed that all systems reached equilibrium after the initial 10 ns, with only minor fluctuations observed throughout the trajectory.” This statement is clear but lacks quantitative support; it would be clearer to specify approximate RMSD ranges, for example: “The RMSD profiles indicated that all systems reached equilibrium after approximately 10 ns, stabilizing around ~X–X Å with only minor fluctuations throughout the remaining trajectory.” Similarly, “The apo protein displayed the lowest RMSD values, indicating inherent structural stability in the unbound state.” could be revised to “The apo protein displayed slightly lower RMSD values compared to the ligand-bound systems, suggesting greater structural stability in the unbound state.” The sentence “Both ligand-bound systems exhibited slightly higher RMSD values, consistent with conformational rearrangements upon ligand binding.” could be softened to “Both ligand-bound systems exhibited slightly higher RMSD values, which may reflect conformational adjustments associated with ligand binding.” In addition, “Importantly, the test compound maintained a relatively stable RMSD with moderate deviations, comparable to the standard, suggesting stable binding without significant structural destabilization.” could be simplified by removing unnecessary emphasis. Finally, “Residue-level flexibility was assessed through RMSF analysis, which demonstrated similar overall patterns across the three systems.” could be clarified for precision.

**Response:**
We thank the reviewer for the positive evaluation of our revised manuscript and for the helpful editorial suggestions. We have carefully implemented all recommended corrections to improve clarity and precision. Specifically, grammatical issues were corrected (e.g., “Dysbiosis, an imbalance…” and “50% of studies”), and several sentences in the molecular dynamics section were revised to improve clarity and scientific accuracy. Where appropriate, we incorporated more precise wording and quantitative descriptions of the RMSD and RMSF results to better reflect the simulation outcomes (see S2_file). These revisions have improved the readability and precision of the manuscript while maintaining the intended interpretation of the computational analyses. All corresponding changes have been incorporated in the revised manuscript.

**Reviewer #3**

**Comment:**
The topic is relevant, and the attempt to combine a systematic review and meta-analysis with in silico work is interesting. The pooled analyses suggest modest reductions in fasting plasma glucose and HbA1c, but the interpretation should remain cautious given the substantial heterogeneity reported across studies and the variability in the included interventions, comparators, and trial durations.

A main issue is that the clinical part of the manuscript combines probiotics, synbiotics, berberine, and some combination interventions into the same pooled analyses, while the mechanistic section focuses only on berberine and α-glucosidase. As written, the mechanistic findings do not fully explain the broader pooled clinical results. The eligibility criteria and included studies also need to be presented more consistently, since some trials appear to involve co-interventions or mixed comparators. The statistical methods require clearer justification for pooling clinically diverse studies, especially given the high heterogeneity. The manuscript would also benefit from clearer explanation of how heterogeneity was handled and from more cautious wording in the Discussion and Conclusion.

**Response:**
We thank the reviewer for the thoughtful and constructive feedback, which helped improve the clarity and methodological transparency of the manuscript. Several revisions were made to address these points.

First, we clarified the relationship between the clinical meta-analysis and the mechanistic computational analyses. The Discussion section now explicitly states that the molecular docking and molecular dynamics simulations focus specifically on berberine–α-glucosidase interactions and should be interpreted as exploratory mechanistic insights rather than explanations for the broader pooled clinical effects observed across probiotics, synbiotics, and berberine interventions.

Second, we improved the description of the eligibility criteria and study characteristics in the Methods section to clarify how studies with co-interventions and mixed comparators were handled. The revised text now specifies that trials were included when intervention effects could be evaluated relative to comparable background therapy.

Third, we strengthened the statistical methods section by providing clearer justification for pooling these interventions. The manuscript now explains that probiotics, synbiotics, and berberine were analyzed together to estimate an overall adjunctive effect on glycemic outcomes, while acknowledging that these interventions have distinct biological mechanisms.

Fourth, we expanded the discussion of heterogeneity. Additional explanations have been included to highlight that the high I² values likely reflect variability in intervention type, probiotic strain composition, berberine dose and formulation, treatment duration, baseline glycemic control, concomitant therapies, and participant characteristics.

Finally, we further moderated the wording in the Discussion and Conclusion sections to emphasize that the observed reductions in fasting plasma glucose and HbA1c are modest and should be interpreted cautiously. The manuscript now clearly states that these interventions should be considered adjunctive strategies rather than replacements for established pharmacological therapies, and that clinical use should be guided by individualized patient assessment and established diabetes management guidelines.
