# Supplementary material for: Probiotics, synbiotics and berberine in Type 2 diabetes mellitus: A systematic review, meta-analysis, and molecular dynamics simulation study
Source: PLoS One. 2026 May 29;21(5):e0348907. doi: 10.1371/journal.pone.0348907 (PMC13221027; doi:10.1371/journal.pone.0348907)
Supplement: Attachment — Submitted filename: Response to Reviewer.docx [file pone.0348907.s008.docx]

**Response to Reviewer #3**

We sincerely thank the reviewer for the careful evaluation of our revised manuscript and for acknowledging the improvements in clarity, structure, and overall presentation. We particularly appreciate the recognition that the interpretation of the modest clinical effects and the presence of substantial heterogeneity are now more appropriately reflected.

We have carefully considered your remaining concern regarding the interpretation and framing of the relationship between the clinical meta-analysis and the in silico findings. In response, we have made the following revisions:

1. **Further clarification of the distinction between clinical and computational components**
   We have revised the Discussion to more explicitly emphasize that the in silico analyses are berberine-specific and exploratory**,** and do not represent mechanistic explanations for the broader pooled clinical effects observed across probiotics, synbiotics, and combination interventions. Additional clarifying statements have been inserted to clearly delineate these components and avoid any implication of a direct mechanistic linkage.
2. **Strengthening of cautious interpretation throughout the manuscript**
   The language in both the Discussion and Conclusion has been further refined to ensure that the computational findings are consistently presented as supportive, hypothesis-generating molecular insights, rather than causal or explanatory evidence for the overall clinical outcomes. We now explicitly state that these analyses are limited to a specific molecular interaction (berberine–α-glucosidase) and do not capture microbiota-mediated or systemic mechanisms underlying glycemic regulation.
3. **Revision of the Conclusion to avoid overstatement**
   The Conclusion has been updated to reinforce a cautious interpretation, clearly indicating that:
   - The observed clinical effects are modest and heterogeneous
   - The computational findings are limited in scope and specificity
   - No direct mechanistic inference should be drawn between the in silico results and the pooled clinical outcomes

These revisions ensure that the manuscript maintains appropriate scientific rigor and avoids overinterpretation of the combined clinical and computational findings.

We believe that these changes fully address the reviewer’s concerns and further strengthen the clarity, balance, and interpretability of the manuscript. We thank the reviewer for their valuable suggestions, which have helped improve the quality of our work.
